# Aqueous Two‐Phase Submicron Droplets Catalyze DNA Nanostructure Assembly for Confined Fluorescent Biosensing

**DOI:** 10.1002/advs.202417287

**Published:** 2025-04-15

**Authors:** Xiaoman Duan, Siyi Duan, Zhaoyu Han, Haoyue Lv, Haozhen Yu, Biwu Liu

**Affiliations:** ^1^ Institute of Analytical Chemistry and Instrument for Life Science The Key Laboratory of Biomedical Information Engineering of Ministry of Education School of Life Science and Technology Xi'an Jiaotong University Xi'an Shaanxi 710049 P. R. China

**Keywords:** aqueous two‐phase system, crowding effect, DNA assembly, submicron droplets

## Abstract

Membraneless organelles (MLOs) are fundamental to cellular organization, enabling biochemical processes by concentrating biomolecules and regulating reactions within confined environments. While micrometer‐scale synthetic droplets are extensively studied as models of MLOs, submicron droplets remain largely unexplored despite their potential to uniquely regulate biomolecular processes. Here, submicron droplets are generated by a polyethylene glycol (PEG)/dextran aqueous two‐phase system (ATPS) as a model to investigate their effect on DNA assembly in crowded environments. The findings reveal that submicron droplets exhibit distinct advantages over microdroplets by acting as submicron catalytic centers that concentrate DNA and accelerate assembly kinetics. This enhancement is driven by a cooperative mechanism wherein global crowding from PEG induces an excluded volume effect, while local crowding from dextran provides weak but nonspecific interactions with DNA. By exploiting both the confinement and phase properties of submicron droplets, a rapid and sensitive assay is developed for miRNA detection using confined fluorescent readouts. These findings highlight the unique ability of submicron droplets to amplify biomolecular assembly processes, provide new insights into the interplay between global and local crowding effects in cellular‐like environments, and present a platform for biomarker detection and visualization.

## Graphical abstract

Submicron droplets, formed by a polyethyleneglycol (PEG)/dextran aqueous two‐phase system·(ATPS), can act as catalytic centers that concentrate DNA and accelerate assembly kinetic in crowded environments. It highlights a cooperative mechanism wherein global crowding from PEG induces an excluded volume effect, while local crowding from dextran provides weak but nonspecific interactions with DNA. Additionally, the study develops a rapid and sensitive assay for miRNA detection using confined fluorescent readouts.

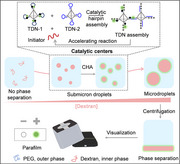

## 1 Introduction

Membraneless organelles (MLOs) are vital for organizing subcellular processes, serving as dynamic compartments that concentrate biomolecules, regulate biochemical reactions, and isolate specific functions without a membrane.^[^ ^1^ ^]^ These biomolecular condensates facilitate ribosome biogenesis, RNA transcription, and DNA repair.^[^ ^2^ ^]^ MLOs vary significantly in size, ranging from 0.1 to 3 µm,^[^ ^3^ ^]^ through rapid condensation and dissolution, as well as fusion and wetting.^[^ ^1d^ ^]^ For instance, nucleoli, Cajal bodies, and nuclear speckles can dynamically alter their size, number, and composition over time, reversibly forming and disappearing during cellular activities such as mitosis.^[^ ^4^ ^]^ The aberrant sizes of condensates have been linked to diseases,^[^ ^5^ ^]^ including neurodegenerative disorders and cancer. For example, soluble tau protein, a key pathogenic factor in Alzheimer's disease, undergoes a phase transition from liquid droplets to gel‐like condensates during maturation, ultimately assembling into large‐sized tau aggregates.^[^ ^6^ ^]^ This underscores the importance of understanding how the size and physicochemical environments of condensates influence crucial biomolecular processes, particularly in crowded intracellular settings.

Synthetic biomolecular condensates formed through liquid–liquid phase separation provide a useful platform for modeling and studying compartmentalization.^[^ ^7^ ^]^ Both associative (e.g., coacervates) and segregative (e.g., aqueous two‐phase systems, ATPS) phase separation have produced versatile membrane‐free droplets that facilitate the investigation of biological processes such as enzymatic activity,^[^ ^8^ ^]^ protein folding,^[^ ^9^ ^]^ and RNA catalysis.^[^ ^10^ ^]^ However, most existing studies focus on microscale droplets, leaving a critical gap in understanding the unique dynamics and biological functions of submicron droplets.^[^ ^11^ ^]^ Observations from in situ liquid transmission electron microscopy have revealed nanoscale protein clusters, suggesting that submicron droplets may govern distinct mechanisms for reaction acceleration and regulation.^[^ ^12^ ^]^ However, the role of submicron droplets in modulating biomolecular reactions remains poorly understood.

Herein, we employ the ATPS as a model system to investigate the role of submicron droplets in the assembly of DNA nanostructures. DNA assembly is essential for various cellular functions, including replication, gene expression, and division. It also plays a foundational role in various biomedical and nanotechnology applications, such as cancer diagnosis,^[^ ^13^ ^]^ organelle interference,^[^ ^14^ ^]^ controlled delivery,^[^ ^15^ ^]^ and immunotherapy.^[^ ^16^ ^]^ Using the catalytic hybridization assembly (CHA) of DNA tetrahedrons (TDNs) as our model reaction,^[^ ^14^, ^16^, ^17^ ^]^ we demonstrate that DNA assembly is significantly accelerated within submicron droplets. We reveal that this enhancement results from a cooperative effect in which global PEG crowding induces an excluded volume effect while local dextran crowders facilitate nonspecific interactions with DNA. Furthermore, we develop a rapid platform for biomarker visualization using confined fluorescent readouts, achieving high sensitivity and selectivity. These findings highlight the role of submicron droplets in promoting biomolecular assembly and offer insights into the interplay of global and local crowding effects in cellular‐like environments.

## 2 Result and Discussion

### 2.1 Droplet Formation and Size Regulation

We utilized a typical ATPS composed of neutral polymers, poly(ethylene glycol) (PEG), and dextran, to generate droplets of varying sizes by adjusting the molecular weights and concentrations of both polymers. The PEG/dextran environment allows us to circumvent the complexities of charge interactions commonly found in polyelectrolyte coacervates. The reversible bulk phase separation in ATPS also facilitates fluorescent assays in biosensing applications. Notably, DNA TDNs preferentially partition into the dextran‐rich phase, making them ideal for probing local droplet environments.^[^ ^18^ ^]^

**Figure 1:**
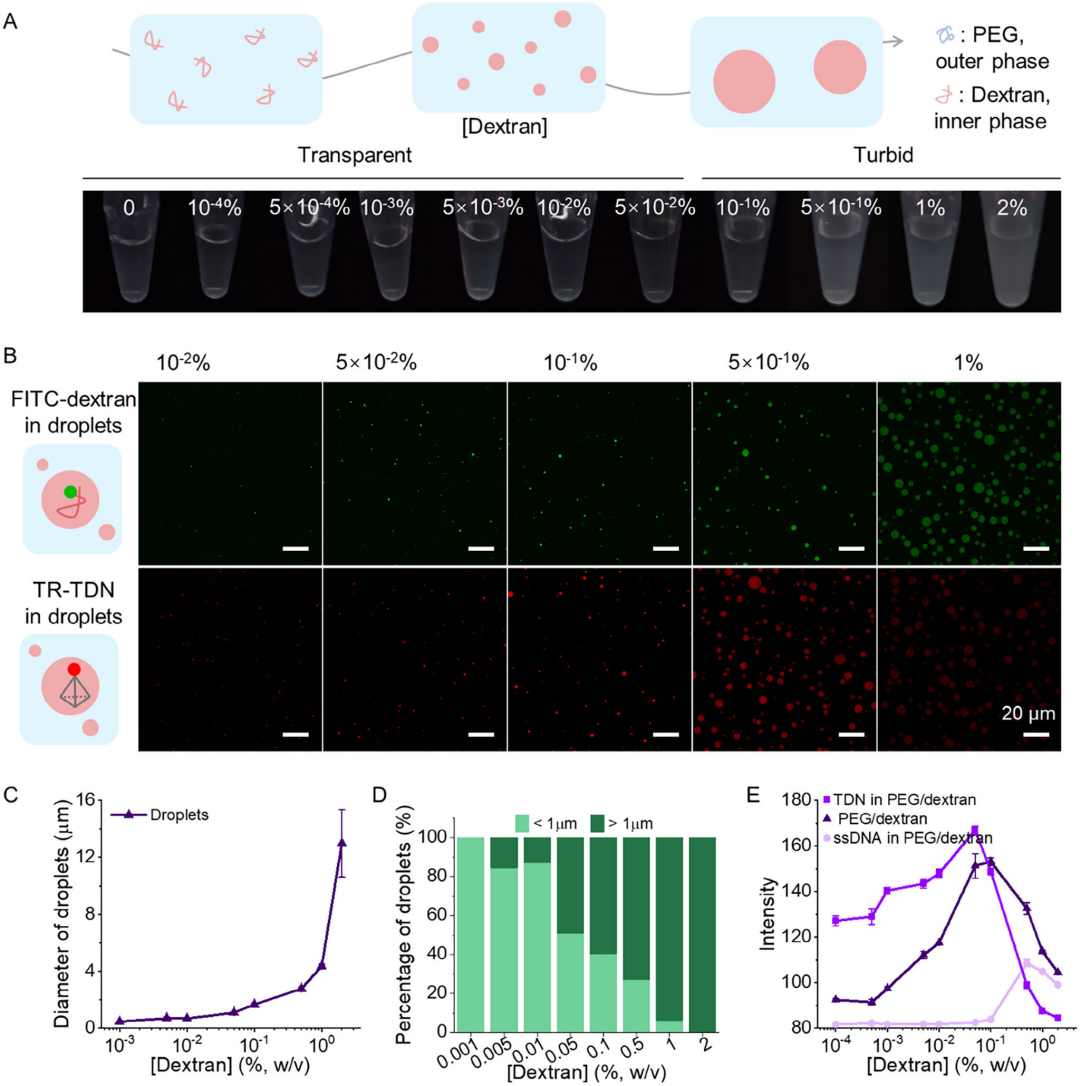
Formation and properties of submicron droplets in ATPS. A) Schematic and optical images showing droplet formation in the PEG/dextran ATPS system at varying dextran 70k concentrations (top row) with a constant 10% PEG 20k. B) Confocal fluorescence images of PEG/dextran droplets showing the partitioning behavior of tetrahedral DNA nanostructures (TDNs) (50 nm) as a function of dextran concentration. C) Average droplet diameter as a function of dextran 70k concentration in the presence of 10% PEG 20k. D) Droplet size distribution at dextran concentrations ranging from 10^−3^% to 2% with a constant 10% PEG 20k. E) Quantitative fluorescence intensity analysis of ssDNA and TDN within droplets using ImageJ software. Data in C,E) are shown as the mean ± SD (*n* = 3).

To explore the droplet formation in the PEG/dextran ATPS, we mixed PEG (20k, 10%, w/v) with dextran of varying concentrations (70k, 0%, 10^−4^%, 5 × 10^−4^%, 10^−3^%, 5 × 10^−3^%, 10^−2^%, 5 × 10^−2^%, 10^−1^%, 5 × 10^−1^%, 1%, and 2%, w/v) in buffer A (10 mm HEPES, pH 7.6, 150 mm NaCl, 5 mm MgCl_2_) (**Figure** **1**A). When the dextran concentration exceeded 0.1%, the solution transitioned to a turbid state, indicative of macroscale phase separation. Furthermore, confocal laser scanning microscopy (CLSM) imaging revealed that the droplets were rich in dextran (Figure S1, Supporting Information) and exhibited typical liquid‐like properties, such as fusion (Figure S2A, Supporting Information). Moreover, fluorescence recovery after photobleaching (FRAP) experiments verified the mobility of FITC‐dextran within the droplets (Figure S2B, Supporting Information). While such higher dextran concentrations are typically used in ATPS, we observed abundant droplets with submicron sizes at lower dextran concentrations (10^−3^%, 5 × 10^−3^%, 10^−2^%, and 5 × 10^−2^%) (Figure 1B, top panel; Figure S3, Supporting Information). Droplet size depends on the dextran concentration, with a critical transition observed at 5 × 10^−2^% dextran (Figure 1C,D; Figure S4, Supporting Information). These results clearly demonstrate that droplet size within the PEG/dextran system can be finely regulated from submicron to microscale by adjusting the dextran concentration.

Next, we investigated the partitioning behavior of DNA TDNs into droplets by labeling one corner of the TDN with a fluorescent dye Texas Red (TR‐TDN, Table S1, Supporting Information). As dextran concentration increased, the fluorescence intensity of TR‐TDN gradually increased, peaking at 5 × 10^−2^% dextran (Figure 1B, bottom panel; Figures S5 and S6, Supporting Information). In contrast, comparable single‐stranded DNA (ssDNA) exhibited minimal fluorescence at higher dextran concentrations, highlighting the specific capability of TDNs for efficient partitioning (Figure S7, Supporting Information). Furthermore, an increase in overall DNA concentration corresponded with enhanced DNA capture in larger droplets (Figure S8, Supporting Information).

To better understand the local environments of droplets, we analyzed the fluorescence intensity of FITC‐dextran (10^−2^%) and DNA TDNs in the PEG/dextran ATPS. For FITC‐dextran, fluorescence intensity within droplets increased with total dextran concentration, peaking at 5 × 10^−2^% (Figure 1E). Beyond this concentration, further droplet growth led to a dilution of FITC‐dextran, resulting in a decrease in fluorescence intensity (Figure 1B, top panel). Interestingly, this critical dextran concentration corresponds to the transition point where droplet size shifts from submicron to microscale (Figure 1D).

Together, these results reveal that submicron droplets formed within PEG/dextran ATPS systems and exhibit a strong capacity for capturing DNA TDNs. The critical dextran concentration (5 × 10^−2^%) serves as a transition point that optimizes both droplet size and local biomolecular concentrations.

### 2.2 Droplet Size‐Dependent Acceleration of DNA Assembly

**Figure 2:**
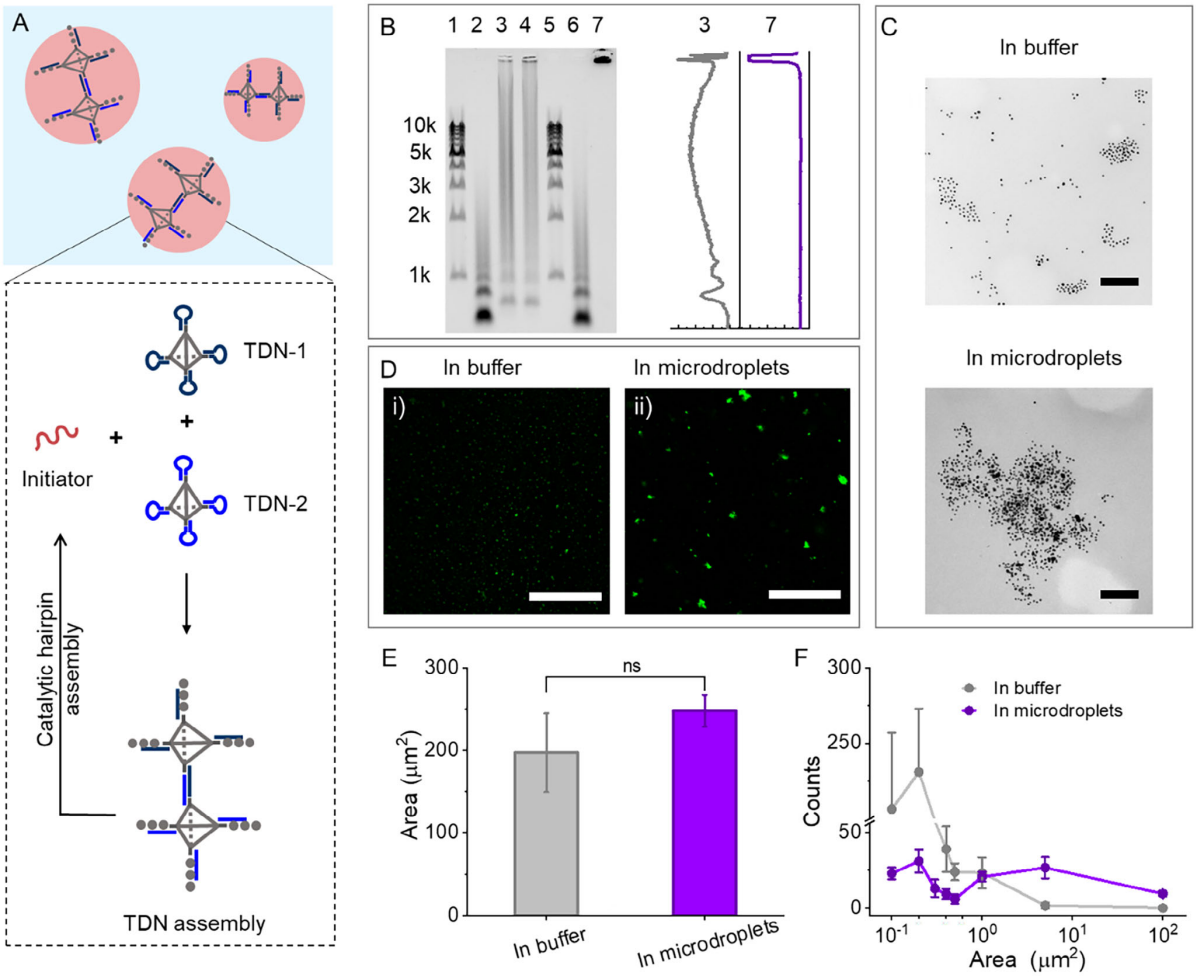
Characterization of DNA TDNs assembly in buffer and microdroplets. A) Schematic illustrating the assembly of DNA TDNs in microdroplets. B) Gel electrophoresis of DNA TDNs and assemblies after introducing Initiator (1 µm) to TDN‐1 and TDN‐2 (1 µm for each) for 1 h in the buffer and microdroplets. C) TEM images and D) CLSM images of DNA assemblies in (i) buffer A and (ii) microdroplets after a 4 h reaction. The concentrations of TDNs were 20 and 100 nm for TEM and CLSM imaging, respectively, with an equal molar Initiator used. E) Area and F) size distribution statistics of TDN assembly in CLSM images. Data are shown as the mean ± SD (*n* = 3). Statistical significance was calculated via unpaired Student's *t*‐tests. “ns” indicated not significant. The scale bars in C) are 200 nm, and in D) are 25 µm.

Next, we investigated how droplet size influences the assembly of DNA nanostructures. We hypothesized that droplets act as catalytic centers by concentrating reactants within confined environments, thereby enhancing DNA assembly. To test this, we employed a catalytic hairpin assembly (CHA) reaction initiated by a 22‐mer DNA oligo (referred to as the Initiator). The Initiator triggered the hybridization between two metastable hairpins (H1 and H2) tethered to TDNs (**Figure** **2**A; Figure S9, Supporting Information). During the CHA cycle, the Initiator hybridizes with the toehold of H1 on TDN‐1, unfolding H1 to expose its loop structure. This loop is subsequently hybridized with a complementary sequence on H2 attached to TDN‐2, facilitating the linkage of TDN‐1 and TDN‐2. After H1 unfolds, the Initiator is released, allowing for repeated cycles of the reaction and driving the formation of large DNA superstructures (Figure S10, Supporting Information).^[^ ^19^ ^]^

To examine the effect of ATPS, the CHA reaction products were first visualized via agarose gel electrophoresis, comparing buffer‐only conditions with the ATPS (10% PEG 20k and 1% dextran 70k) after 1 h (Figure 2B). In buffer A, a diffused band was observed on the gel (lane 3), indicating a broad distribution of DNA assembly sizes. In contrast, when the reaction was conducted in ATPS microdroplets, the reaction products predominantly accumulated in the gel wells, with no distinct band visible in the lanes, suggesting the formation of significantly larger DNA aggregates (lane 7). To eliminate potential interference from matrix components, the PEG‐rich phase was carefully removed after bulk phase separation (3 min at 3500 rpm). Control experiments confirmed that residual dextran did not influence TDN migration (lane 4).

Further characterization using transmission electron microscopy (TEM) and fluorescence imaging provided insights into the TDN assemblies. To enable high‐contrast visualization of TDN assemblies, 13‐nm gold nanoparticles (AuNPs) were site‐specifically conjugated to a single vertex of the TDN through DNA hybridization (Figure S11A,B, Supporting Information). TEM imaging revealed assemblies with an average size of ca. 900 nm with the assembled TDN architectures exhibiting enhanced structural compactness when in microdroplets, compared to smaller and more relaxed structures (ca. 130 nm) in buffer A (Figure 2C; Figure S11, Supporting Information). Fluorescence imaging with a DNA staining dye (SYBR Green I) and CLSM confirmed these observations. While TDNs alone were not readily detectable due to resolution limitations, scattered fluorescent dots emerged in buffer reactions following CHA, whereas much larger fluorescent aggregates formed within microdroplets (Figure 2D; Figures S12 and S13, Supporting Information).

Quantitative analysis of multiple images revealed a comparable total assembly area between buffer and ATPS, suggesting a similar number of hairpins participated in both conditions (Figure 2E; Figures S13 and S14, Supporting Information). However, the size distributions differed markedly. In buffer A, the majority of assemblies were small, with micron‐sized aggregates comprising just 0.3% of assemblies, occupying ≈5% of the total volume (Figure S13, Supporting Information). In contrast, over 26% of assemblies in ATPS were micron‐scale, accounting for nearly 96% of the total assembly volume (Figure S14, Supporting Information). The disparities in size and distribution align with the gel electrophoresis results, emphasizing the ability of ATPS to influence and enhance DNA assembly.

**Figure 3:**
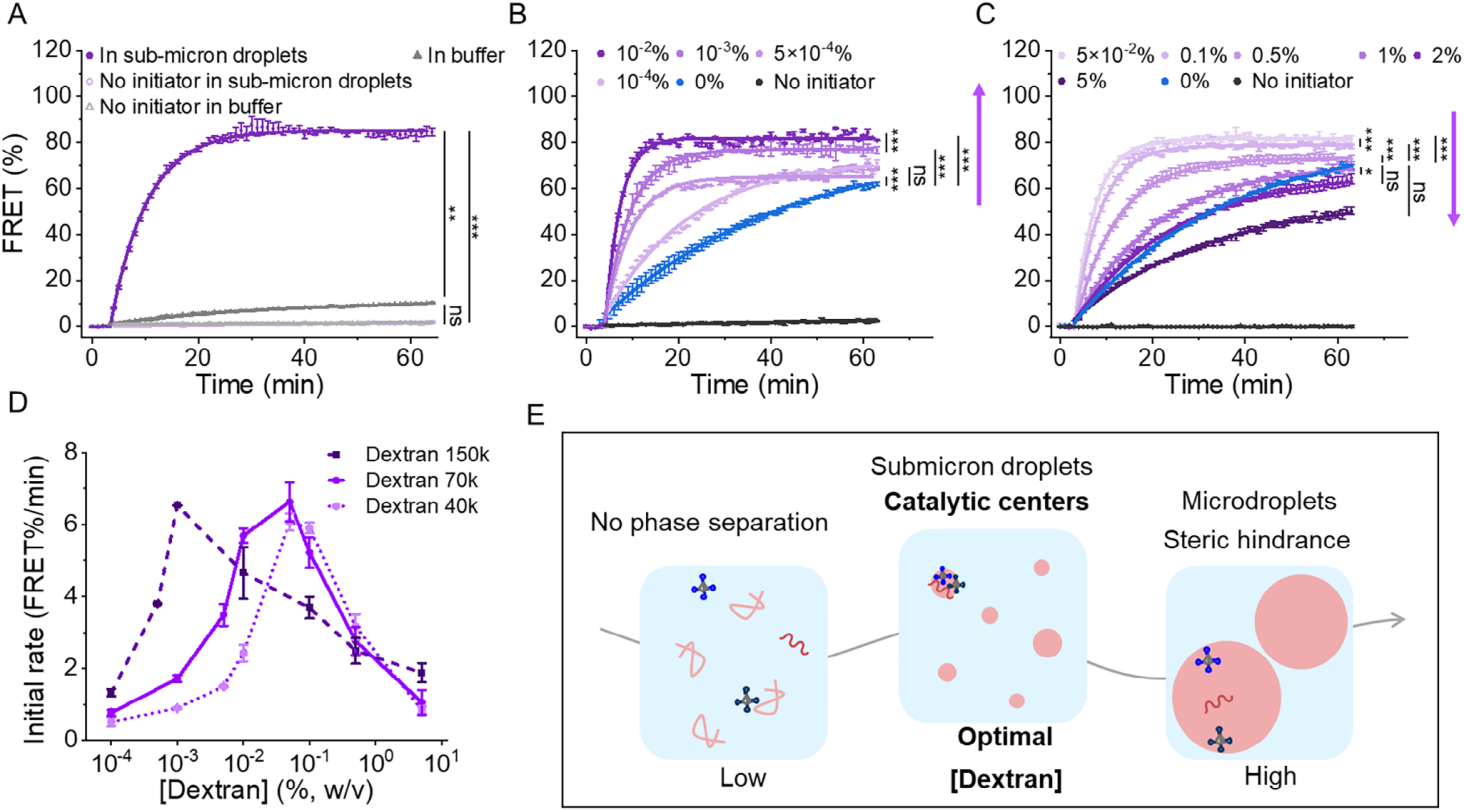
TDN assembly was facilitated by submicron droplets. A) Comparison of TDN assembly (5 nm) in buffer A and the sub‐micron droplets (10% PEG 20k/0.05% dextran 70k). Kinetics of DNA assembly with constant PEG (20k, 10%) as a function of dextran 70k concentrations from B) 10^−4^% to 10^−2^% and C) 5×10^−2^% to 5%. D) The initial rates as a function of dextran (40, 70, and 150k) concentration with 10% PEG 20k. E) Scheme showing the TDN assembly in a mixed crowding environment with different dextran concentrations. Data are shown as the mean ± SD (*n* = 3). Statistical significance was calculated via one‐way ANOVA with Tukey's test: *p < 0.05, **p < 0.01, and ***p < 0.001; “ns” indicated not significant.

Next, we sought to explore how droplet size in ATPS affects DNA assembly dynamics. To this end, reaction rates were monitored using hairpins H1 and H2 labeled with a Förster resonance energy transfer (FRET) pair (FAM and TAMRA, Table S1, Supporting Information) under conditions where submicron droplets are predominant (0.05% dextran and 10% PEG). Without the Initiator, the FRET signal remained stable (**Figure** **3**A). After introducing the Initiator (5 nm), a strong FRET signal emerged rapidly, reaching a plateau within 30 min in submicron droplets, while the signal increased slowly in buffer A. TEM images revealed the formation of larger and even more densely packed assemblies within sub‐micron droplets (10% PEG 20k and 0.05% dextran 70k) (Figure S15, Supporting Information), indicating the superior roles of submicron droplets.

We then systematically varied the dextran concentrations in the ATPS from 10^−4^% to 5%, while maintaining PEG 20k at a constant 10% (Figure 1C). Even at low dextran concentrations (10^−4^%), the reaction rate increased compared to the buffer system, reaching a maximum at 0.05% dextran (Figure 3B). Notably, at this concentration, ATPS droplets predominantly remained in the submicron range, creating optimal conditions for DNA assembly. Beyond this critical concentration, reaction rates steadily declined with higher dextran concentrations, eventually becoming inhibited at 5% dextran (Figure 3C). These results suggest that submicron droplets produce optimal reaction conditions for CHA by concentrating reactants, while larger microdroplets inhibit assembly due to steric hindrance and reduced localized concentrations of reactants.

To further validate the role of droplet size, we examined how dextran molecular weight influenced the CHA reaction (Figure 3D). Higher molecular weight dextran (150k) shifted the optimal dextran concentration to an even lower range. For instance, the critical concentration shifted to 10^−3^% for dextran 150k, compared to 0.05% for dextran 40 and 70k (Figures S16–S19, Supporting Information). Importantly, varying the concentration of TDNs did not alter the critical dextran concentration, indicating that the ATPS environment, rather than TDN concentration, governs reaction efficiency (Figure S20, Supporting Information). Together, our results demonstrate that submicron droplets in ATPS are more efficient in facilitating DNA assembly.

### 2.3 Mechanistic Investigations

**Figure 4:**
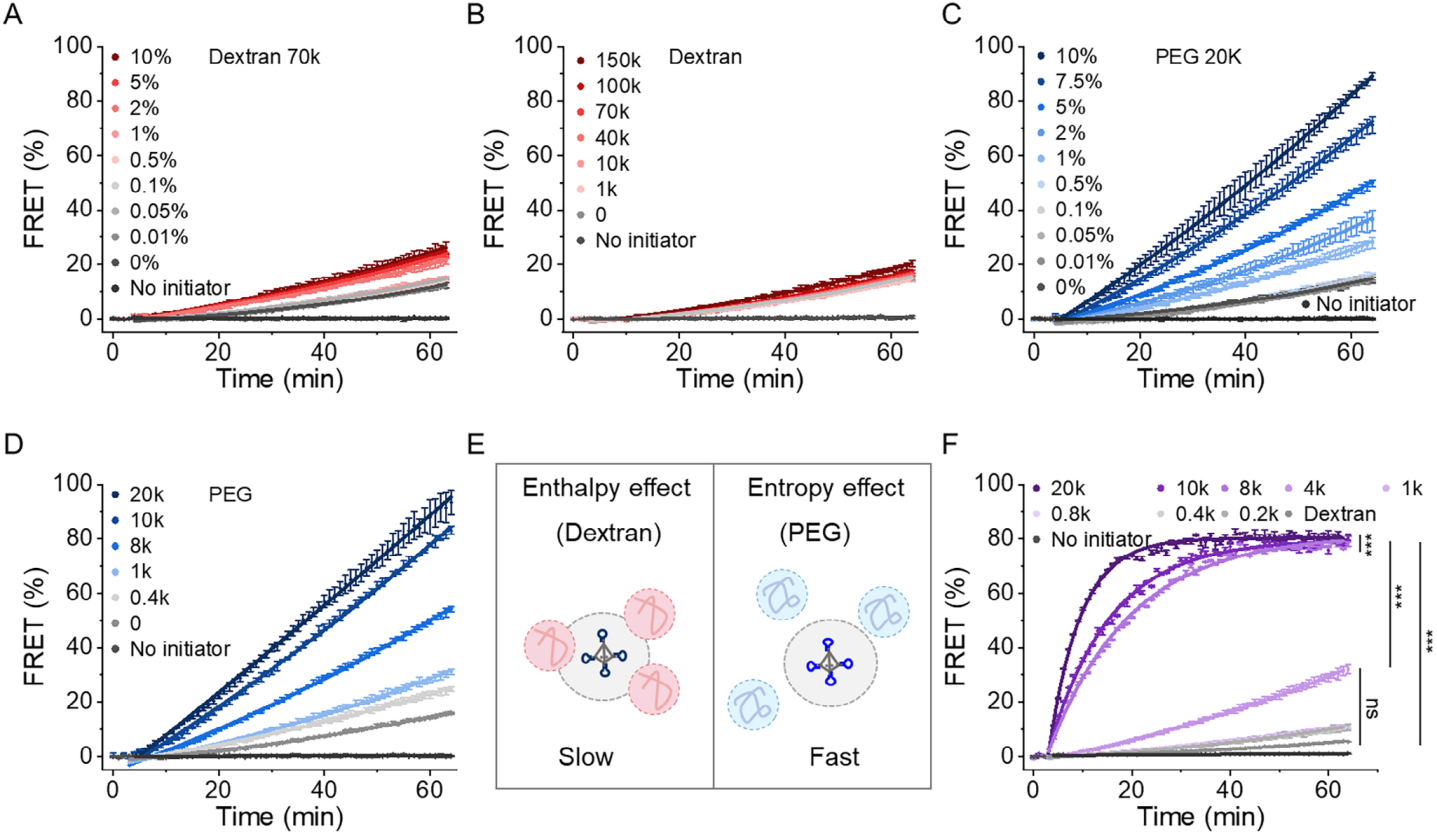
Cooperative crowding effect of global PEG and local dextran on the reaction kinetics. Kinetics of DNA assembly as a function of A) dextran 70k concentration, B) dextran molecular weights (10%, 1, 10, 40, 70, 100, and 150k), C) PEG 20k concentration, D) PEG molecular weights (10%, 0.4, 1, 8, 10, and 20k) and F) PEG molecular weights (10%) with constant dextran (70k, 0.1%). TDNs and Initiators are 5 nm. E) Schematic model for the effect of PEG (entropy effect) and dextran (enthalpy effect) on accelerating DNA TDN assembly. Data are expressed as the mean ± SD (*n* = 3). Statistical significance was calculated via one‐way ANOVA with Tukey's test: ***p < 0.001; “ns” indicated not significant.

Having established the role of submicron droplets, we next sought to explore the underlying mechanisms of the reaction processes. Within the ATPS, DNA TDNs are trapped in the dextran‐rich phase. To determine whether this local trapping drives the enhanced reactions, we tested TDN assembly in a pure dextran solution (**Figure** **4**A,B). Increasing the dextran concentration from 0 to 10% resulted in only a modest 1.8‐fold increase in the initial reaction rate (Figure 4A). This enhancement is significantly lower than the ca. 16‐fold increase in ATPS with 0.05% dextran (Figure 3A). Additionally, the dextran of different MWs revealed only minor improvements (Figure 4B).

Next, we considered that residual PEG in the dextran‐rich phase might account for the observed rate enhancement. To test this, we pre‐mixed PEG and dextran, used centrifugation to separate the phases, and used the dextran‐rich phase as the reaction matrix. The reaction kinetics in this isolated dextran‐rich phase were comparable to those seen in pure dextran solutions (Figure S21, Supporting Information). Collectively, these results demonstrate that the locally crowded dextran‐rich environment does not explain the observed acceleration of DNA assembly.

Having ruled out dextran as the primary driver of enhanced assembly, we shifted our focus to PEG, a well‐studied macromolecular crowding agent used as a mimetic for intracellular crowding.^[^ ^20^ ^]^ Increasing the concentration of PEG 20k significantly enhanced TDN assembly rates, up to a ≈16‐fold increase (Figure 4C). Moreover, the reaction rate was further accelerated with higher MW PEGs (Figure 4D; Figure S22, Supporting Information). However, PEG 35k and PEG 100k were not used in subsequent experiments due to their high viscosity, which introduced challenges in pipetting and achieving reproducible experimental conditions.

The stark contrast between PEG and dextran underscores their divergent reaction mechanisms in crowding. PEG acts as a “global crowder,” creating a densely crowded environment that promotes DNA hybridization and assembly through an excluded volume effect (entropic effect). In contrast, the relatively weak promotion by dextran is likely due to nonspecific enthalpic interactions (e.g., binding to nucleic acids) that counteract the expected acceleration effect of crowding (Figure 4E). These findings align with single‐molecular FRET studies by Sung and Nesbitt.^[^ ^21^ ^]^ They demonstrated that PEG behaves as a “true” entropic crowder, whereas dextran (and Ficoll) exhibits entropic‐enthalpic interactions that diminish net crowding efficacy. The observed entropy/enthalpy compensation results in a diminished crowding effect by dextran.

To confirm this hypothesis, we evaluated additional crowding agents. Ficoll, a neutral polysaccharide, produced similar results to dextran, with only modest reaction enhancements (Figure S23, Supporting Information). In contrast, poly(diallyl dimethylammonium chloride) (PDDA), a positively charged polymer, exhibited concentration‐dependent effects: at low concentrations, it enhanced TDN assembly by electrostatically screening DNA charges; however, at high concentrations, it inhibited assembly due to the electrostatic attraction between the polymer and DNA (Figure S24, Supporting Information). These results establish that while charged or specific‐binding polymers can influence TDN assembly, true crowding effects induced by PEG play a dominant role in enhancing reaction kinetics.

To further dissect the mechanisms underlying DNA TDN assembly in ATPS, we fixed dextran concentration at 0.1% and varied the MW of PEG from 0.2 to 20k. We observed a dramatic ca. 70‐fold increase in the initial reaction rate with increasing PEG MW (Figure 4F). These observations strongly support the synergy between global PEG crowding and local droplet confinement drives the accelerated DNA assembly. Specifically, droplets act as catalytic centers by concentrating DNA TDNs within a confined environment, while PEG provides a global crowding effect that reduces the entropy of the system and enhances the hybridization kinetics (Figure 4E).

We next investigated whether these mechanisms apply to other DNA assembly processes, using a variety of reaction designs that produce different structural products. By varying the number of vertices connecting the TDN, we manipulated the products to form TDN dimers, linear chains, or three‐dimensional aggregates (Figures S25 and S26, Supporting Information). In all cases, PEG significantly accelerated assembly rates compared to dextran alone, and the strongest enhancements were seen in the droplet environment. Notably, FRET efficiency analysis at 30 min revealed that the catalytic effect of submicron droplets increased with the number of vertices per TDN unit, underscoring the role of structural complexity in modulating assembly dynamics (Figure S26, Supporting Information). Similarly, TDNs of varying sizes (Figures S27–S29, Supporting Information) exhibited consistent behavior, with PEG and submicron droplets cooperating to promote assembly across diverse DNA systems.

These findings highlight the cooperative mechanisms by which PEG and dextran enhance DNA TDN assembly. While dextran‐rich droplets serve as catalytic centers that concentrate reactants, PEG exerts a global excluded‐volume effect that drives the high‐efficiency hybridization and assembly of DNA nanostructures. This mixed crowding environment creates a system‐intrinsic synergy, where local confinement and global crowding effects combine to facilitate highly efficient reactions.

### 2.4 Visualizing Biomarkers with Confined Readouts

**Figure 5:**
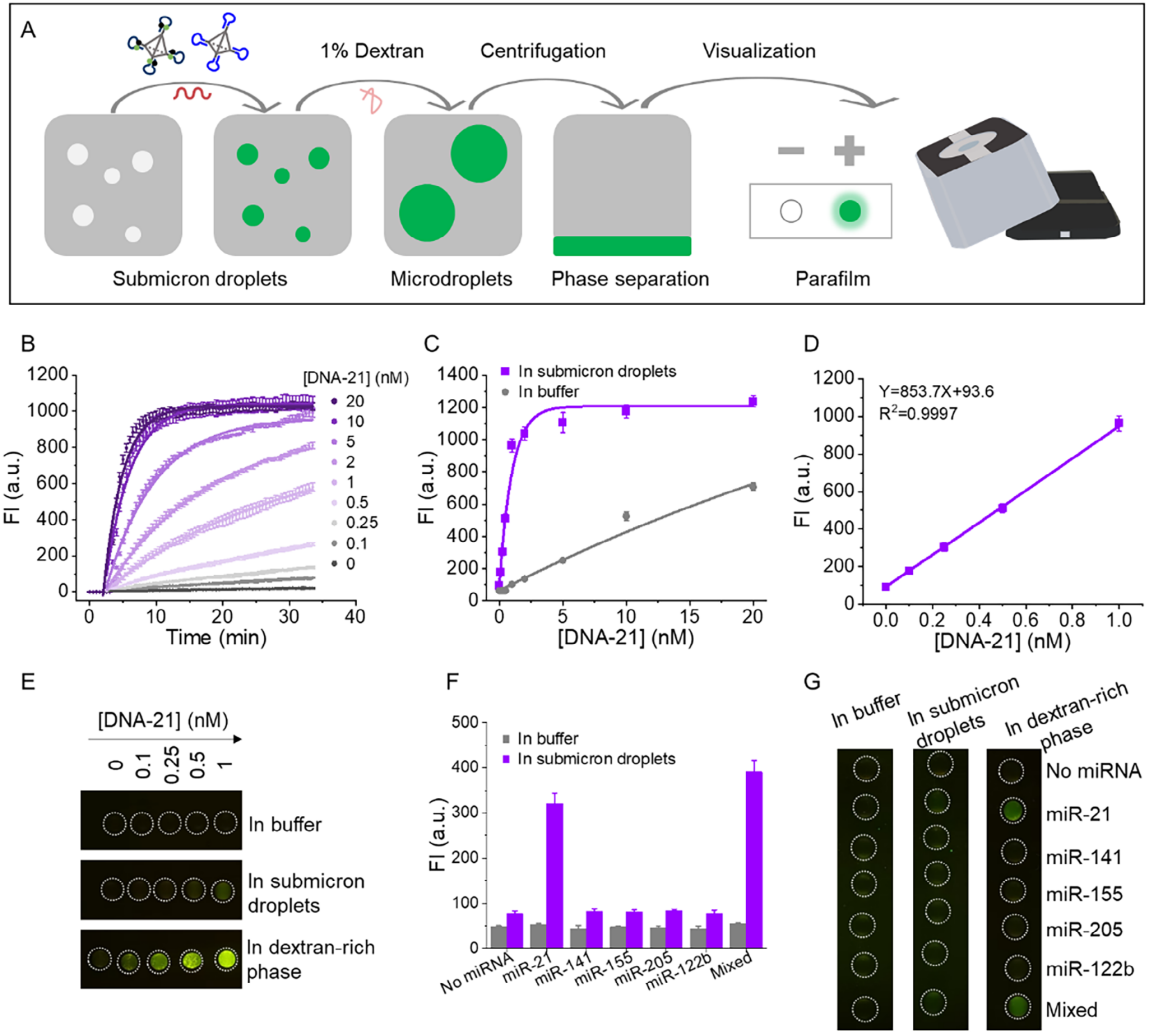
Visualized detection of miRNA‐21 by using an aqueous two‐phase system. A) Schematic illustration of the observation method only with the help of the blue LED excitation. B) Kinetics of target detection at different concentrations (from bottom to top: 0, 0.1, 0.25, 0.5, 1, 2, 5, 10, and 20 nm). TDN‐1‐FAM‐BHQ1 and TDN‐2 were 5 nm in 10% PEG/0.05% dextran ATPS in buffer A. C) Detection calibration curves in buffer A and the submicron droplets (10% PEG 20k/0.05% dextran 70k). D) Linear response for detecting a target in the submicron droplets (10% PEG 20k/0.05% dextran 70k). E) Photographs for visual detection of the target with various concentrations. TDN‐1‐FAM‐BHQ1 and TDN‐2 were 5 nm. The reaction time was 30 min at room temperature. Submicron droplets were composed of 10% PEG 20k and 0.05% dextran 70k. F) Fluorescence intensity and G) images of selectivity of the CHA‐TDN probe in the submicron droplets (10% PEG 20k and 0.05% dextran 70k). The concentration of miRNA was 1 nm. Error bars in B, C, D, and F represent the standard deviation based on three independent measurements.

Lastly, we developed a simplified and efficient method for biomarker visualization using a droplet‐confined readout. This approach combines the accelerated DNA assembly within submicron droplets and the phase separation properties of micrometer‐sized droplets for signal amplification (**Figure** **5**A). To monitor reaction kinetics, we labeled H1 with a FRET pair (FAM and BHQ1) (Table S1; see Supporting Information for details). Following a 30‐minute CHA‐TDN reaction in the submicron droplets (10% PEG 20k/0.05% dextran 70k), the fluorescence intensity of FAM increased. A final concentration of 1% dextran was then introduced to induce phase separation, forming microdroplets that concentrated at the bottom of the tube after centrifugation (3 min, 3500 rpm). The FAM fluorescence signal enriched in the dextran‐rich phase was further amplified. Once the PEG‐rich phase was removed, the dextran‐rich phase was transferred to parafilm, where it could be observed directly under a blue LED lamp.

We first validated the feasibility of detecting miRNA‐21 using a DNA mimic. Without DNA‐21, the hairpin H1 remained closed, resulting in low fluorescence. As increasing amounts of target DNA were added, fluorescence intensities rose correspondingly (Figure 5B). These results demonstrate that crowded submicron droplets act as catalytic reactors, accelerating DNA assembly and amplifying signals. Furthermore, we tested the detection system across varying dextran concentrations (10^−4^% to 5%) within the PEG/dextran system. Reaction rates followed a similar trend (Figure S30, Supporting Information), and the fluorescence intensity in submicron droplets was ca. 24‐fold higher than in buffer due to signal enrichment in the dextran‐rich phase (Figure 5C).

Using this approach, a linear relationship was observed between fluorescence intensity and target concentration in the range of 0.1–1.0 nm (Figure 5D). The limit of detection (LOD), calculated using the 3σ/slope rule, was determined to be 1.9 pm, representing an 18.5‐fold improvement. For comparison, detection under three different environments (buffer, submicron droplets, and the dextran‐rich phase after separation) was analyzed (Figure 5E). In the buffer, no fluorescence was observed. In submicron droplets, fluorescence became detectable only at target concentrations above 0.5 nm. Following the introduction of 1% dextran, fluorescence was significantly enhanced, allowing the detection down to 0.1 nm target, which was further confirmed using semi‐quantitative analysis with ImageJ software (Figure S31, Supporting Information). These results validate the system's ability to reliably and sensitively detect miRNA‐21.

We then evaluated the selectivity of this method by testing its performance with other miRNA families, including miR‐141, miR‐155, miR‐205, and miR‐122b. In the buffer, miRNA‐21 could not be differentiated. However, in submicron droplets, the fluorescence induced by miRNA‐21 was approximately four times higher than that of other miRNAs, highlighting its strong selectivity. Finally, we tested the system's visualization capabilities on parafilm without the need for specialized fluorescence instruments. Using a blue LED lamp, fluorescence induced by miRNA‐21 could be easily observed following signal enrichment in the dextran‐rich phase. In contrast, this fluorescence was too weak to discern in the buffer or submicron droplets alone (Figure 5F,G).

Overall, these results demonstrate the practicality of using ATPS with varying droplet sizes as a convenient and effective visualization tool for miRNA detection. This system combines accelerated DNA assembly and signal enrichment to achieve high sensitivity and selectivity, making it a promising platform for fast and accessible biomarker detection.

## 3 Conclusion

In summary, we have demonstrated the formation of submicron droplets in the PEG/dextran ATPS system and revealed the cooperative roles of local submicron droplets and global PEG crowding effects in facilitating efficient DNA TDN assembly within a mixed crowding environment. Submicron droplets act as catalytic centers, concentrating reactants within submicron compartments and creating a confined environment that accelerates DNA hybridization and assembly. In contrast, global crowders, such as PEG, exert a dominant entropic effect that enhances reaction efficiency by inducing an excluded volume effect. Additionally, we identified that enthalpic interactions between the local crowder (dextran) and DNA occur at an optimal dextran concentration (0.05% dextran 70k with 10% PEG 20k) for assembly, highlighting the fine balance between entropy and enthalpy in these systems.

Our findings offer two key insights into the behavior of mixed crowding environments, such as the cytosol. First, even within compartmentalized systems, global crowding plays a pivotal role in determining reaction kinetics and outcomes. This suggests that macromolecular crowding effects can extend beyond local compartments, influencing molecular behavior within and across these regions. Beyond DNA assembly, recent work shows phospholipid content enriched in droplets could be controlled by the size of biomolecular condensates.^[^ ^22^ ^]^ Second, local compartments, such as submicron droplets or membrane‐less organelles, are likely to have dual roles in biochemical processes. Depending on the balance of cooperative entropy and enthalpy effects, these compartments may promote or inhibit various macromolecular interactions. The assembly of cellulose nanocrystals also requires an optimal concentration. Different PEG/dextran ratios can disrupt the assembly process, leading to ineffective aggregation or undesirable phase behavior.^[^ ^23^ ^]^ These insights highlight the need to consider both global and local crowding effects when studying macromolecular assembly and reaction dynamics. Broadly, our findings provide a framework for understanding the complex mix of crowding effects within cellular environments, offering implications for biochemical regulation, biomolecular assembly, and the design of new synthetic systems for biosensing and diagnostics.

## 4 Experimental Section

### Materials

All DNA samples were purchased from Sangon Biotech (Shanghai, China). Upon arrival, they were dissolved into Milli‐Q water at a concentration of 100 µm and stored in a −20 °C freezer until use. The sequences and modifications of the DNAs are shown in Table S1 (Supporting Information). Sodium chloride and 4‐(2‐hydroxyethyl) piperazine‐1‐ethanesulfonic acid (HEPES) were from Aladdin (Shanghai, China). Magnesium chloride hexahydrate was from Sinopharm (Shanghai, China). Polyethylene glycol (PEG, 0.2, 0.4, 0.8, 1, 4, 8, 10, 20, 35, and 100k), dextran (1, 10, 40, 70, 100, and 150k), Fluorescein 5‐isothiocyanate (FITC)‐dextran (70k) and poly (diallyl dimethylammonium chloride) 35 wt.% solution (< 100k) (PDDA 100k) were purchased from Macklin (Shanghai, China). Ficoll (70k) was purchased from Aladdin (Shanghai, China). Tris‐Borate‐EDTA buffer (TBE) and SYBR Green I were from Beyotime (Shanghai, China). Milli‐Q water was used for all experiments.

### Preparation of DNA TDN

Taking TDN‐1 as an example, DNA oligonucleotides (17‐1, 17‐2, 17‐3, 17‐4, and H1) were mixed in the ratio of 1:1:1:1:4 in Tris‐HCl buffer (10 mm, pH 8) containing MgCl_2_ (50 mm). The mixture was heated to 95 °C for 5 min, followed by a rapid cooling to 4 °C within 30 s. The resulting TDN concentration was 1 µm. TDNs of varying lengths or with different tethered hairpins were prepared using a similar method.

### Assembly of DNA TDNs

The assembly of DNA TDNs was carried out in buffer A (10 mm HEPES, pH 7.6, 150 mm NaCl, and 5 mm MgCl_2_). The concentration of TDNs may vary depending on specific conditions. Typically, TDN‐1 and TDN‐2 (5 nm) were mixed with Initiator (5 nm) in buffer A for the assembly process. To facilitate the assembly of DNA TDNs in a crowded environment, polymers with designed concentrations, molecular weights, and ratios were introduced into buffer A.

### Agarose Gel Electrophoresis

The TDN was analyzed using 3% agarose gel electrophoresis in 1x TBE buffer at a constant voltage of 120 V for 45 min. Gel imaging was performed using a Syngene G: BOX imaging system. For TDN assembly analysis, 0.6% agarose gel electrophoresis was used in 1x TBE buffer at a constant voltage of 60 V for 2 h.

### Assembly of TDN‐AuNPs

First, DNA‐AuNPs were prepared by the freezing‐assisted method.^[^ ^24^ ^]^ The received thiolated DNA samples were directly dissolved in Milli‐Q water without further treatment. Typically, 3 µL of 5HS‐27A‐c12 (100 µm) was added into 100 µL citrate‐AuNPs (13 nm, 10 nm). The mixture was vortexed gently and then placed in a −20 °C freezer for 2 h. After thawing at room temperature for 10 min, 4 µL of 250 mm HEPES buffer (pH 7.6) was added. Finally, the obtained DNA‐AuNPs were centrifuged (14 800 rpm, 15 min) to remove the free DNA, washed with HEPES buffer (10 mm, pH 7.6) twice, and dispersed in HEPES buffer (10 mm, pH 7.6).

TDN‐AuNPs were prepared by mixing the DNA‐AuNPs and TDN (17‐3‐6T12 used) (*Au*/*TDN* = 1/20 ) at 37 °C for 1 h. The mixture also contains 10 mm HEPES (pH 7.6), and 150 mm NaCl. Finally, the obtained TDN‐AuNPs were centrifuged (14 800 rpm, 15 min) to remove the free DNA, and washed with 10 mm HEPES buffer (pH 7.6, containing 150 mm NaCl) two times.

TDN‐1‐AuNPs and TDN‐2‐AuNPs (final 2 nm AuNPs) were mixed in 10 mm HEPES (pH 7.6), 150 mm NaCl without or with PEG/dextran and then Initiator (final 20 nm) was introduced. The reaction was 30 min at 25 °C.

### Transmission Electron Microscope (TEM)

Each solution (7 µL, final 2 nm AuNPs) was deposited on the copper grid and allowed to dry naturally. For the solution containing polymers, centrifugation (14 800 rpm, 15 min) two times was required to avoid polymer interference. Transmission electron micrographs were obtained using thermo scientific Talos L120C G2 (120 kV).

### Confocal Fluorescence Microscopy

Glass coverslips were pre‐cleaned with water and dried with nitrogen. Then, 10 µL of the sample was added to the glass. Fluorescence images were acquired using a confocal microscope with a 63× water immersion objective. FITC was excited with a 488 nm laser line and detected with a 495–545 nm band‐pass filter. The Cy5 channel was excited with a 649 nm laser line and detected with a 655–700 nm band‐pass filter.

### Kinetics of DNA Assembly Using FRET

Typically, 10 µL of DNA TDNs (100 nm) was added into a 96‐well plate containing 90 µL of buffer A with or without additional polymers. The fluorescence intensity at 584 nm (TAMRA) and 525 nm (FAM) under 485 nm excitation was recorded for 3 min, followed by adding 5 µL of Initiator (100 nm). To explore the effect of single polymer concentration, buffer A was mixed with dextran 70k (10^−2^%, 5 × 10^−2^%, 0.1%, 0.5%, 1%, 2%, 5%, and 10%), PEG 20k (10^−2^%, 5 × 10^−2^%, 0.1%, 0.5%, 1%, 2%, 5%, 7.5%, and 10%), Ficoll (10^−2^%, 0.5%, 1%, 2%, 5%, 7.5, and 10%), and PDDA (10^−4^%, 10^−3^%, 10^−2^%, 0.1%, 1%, 5%, and 10%). The effect of single polymer MWs was examined similarly using 10% PEG (0.2, 0.4, 0.8, 1, 4, 8, 10, 20, 35, and 100k) and 10% dextran (1, 10, 40, 70, 100, and 150k).

A mixed PEG and dextran were introduced to buffer A for DNA assembly to explore the effect of mixed crowding. For example, the PEG 20k concentration was fixed (10%), and then the dextran 70k concentration was varied (10^−4^%, 5 × 10^−4^%, 10^−3^%, 10^−2^%, 5 × 10^−2^%, 0.1%, 0.5%, 1%, 2%, and 5%).

### Normalization of FRET Signals

An equal ratio of H1‐FAM and H2‐TAMRA (final 20 nm) was introduced to buffer A. Then the mixture was heated at 95 °C for 5 min and cooled at 4 °C overnight to maximize the hybridization between H1 and H2. The single hairpin was treated in the same way. Subsequently, the fluorescent intensities were collected for the FAM channel (emission: 525 nm) and the TAMRA channel (emission: 584 nm) with 485 nm excitation. The FRET signal (*F*) was calculated based on the ratio:

(1) $$F=F_{584}/F_{525}\;$$

Set *F* of H1‐FAM and H2‐TAMRA after annealing as *F_max_*, and *F* of the single hairpins without hybridization as *F_min_*.

Define *F*_100%_

(2) $$F_{100\%}=F_{max}\;-F_{min}$$

Then, the normalized FRET is as follows:

(3) $$FRET\;\left(\%\right)=\left(F_{t}-F_{0}\right)/F_{100\%}\times 100\%$$

Set the *F* at time “t” to *F_t_*, and the average value of *F* for the first three minutes without adding the Initiator to *F*_0_.

### Data Fitting

The time courses were fitted using a single exponential equation.

(4) $$FRET\;\left(\%\right)=A\left(1-e^{-t/k}\right)+C$$

where *F* is the normalized FRET signal at the time “t”. Then the initial reaction rate (*V)* was calculated by

(5) V=−A/k

Alternatively, for the initial rates in Figures 3B–D and 4A–D,F, the reaction over the initial five minutes was recorded and the slope with linear fittings was used.

### Statistical Analysis

The pre‐processing of data was shown as the “Normalization of FRET Signals” subsection above. The results were all expressed as mean ± standard deviation (variance ± SD). The key experiments have been repeated at least 3 times, quantitated, and subjected to statistical analysis. n and *р* values were indicated in every single figure. Two‐group comparisons were performed using unpaired Student's *t*‐tests. For comparisons between multiple groups, a one‐way analysis of variance (ANOVA) followed by Tukey's post hoc test was performed. Differences between groups were considered statistically significant at *p < 0.05, **p < 0.01, ***p <0.001, and ****p < 0.0001; “ns” indicated not significant. Analysis and graphing were performed using OriginLab, ImageJ, and Adobe Photoshop software.

## Conflict of Interest

The authors declare no conflict of interest.

## Supporting information



Supporting Information

## Data Availability

The data that support the findings of this study are available from the corresponding author upon reasonable request.
