# Supplementary material for: Aqueous Two‐Phase Submicron Droplets Catalyze DNA Nanostructure Assembly for Confined Fluorescent Biosensing
Source: Adv Sci (Weinh). 2025 Apr 15;12(22):2417287. doi: 10.1002/advs.202417287 (PMC12165094; doi:10.1002/advs.202417287)
Supplement: Supplementary file 1 — Supporting Information [file ADVS-12-2417287-s001.docx]

Supporting Information

Aqueous Two-Phase Submicron Droplets Catalyze DNA Nanostructure Assembly for Confined Fluorescent Biosensing

Xiaoman Duan, Siyi Duan, Zhaoyu Han, Haoyue Lv, Haozhen Yu, and Biwu Liu*

# Sequences

Table S1. The sequence of the DNA samples used in this work

| DNA names | Sequences and modifications (from 5’ to 3’) |
| --- | --- |
| TR-T17-1 | (**TR**)TCAACTGCCTGGTGATAAAACGACACTACGTGGGAATCTACTATGGCGGCTCTTC |
| T17-1 | TCAACTGCCTGGTGATAAAACGACACTACGTGGGAATCTACTATGGCGGCTCTTCTTTTTTCCCAGGTTCTCT |
| T17-2 | TTCAGACTTAGGAATGTGCTTCCCACGTAGTGTCGTTTGTATTGGACCCTCGCAT |
| T17-3 | TATCACCAGGCAGTTGACAGTGTAGCAAGCTGTAATAGATGCGAGGGTCCAATAC |
| T17-4 | ACATTCCTAAGTCTGAAACATTACAGCTTGCTACACGAGAAGAGCCGCCATAGTA |
| T26-1 | GCCTGGAGATACATGCACATTACGGCTTTCCCTATTAGAAGGTCTCAGGTGCGCGTTTCGGTAAGTAGACGGGACCAGTTCGCCTTTTTTCCCAGGTTCTCT |
| T26-2 | CGCGCACCTGAGACCTTCTAATAGGGTTTGCGACAGTCGTTCAACTAGAATGCCCTTTGGGCTGTTCCGGGTGTGGCTCGTCGG |
| T26-3 | GGCCGAGGACTCCTGCTCCGCTGCGGTTTGGCGAACTGGTCCCGTCTACTTACCGTTTCCGACGAGCCACACCCGGAACAGCCC |
| T26-4 | GCCGTAATGTGCATGTATCTCCAGGCTTTCCGCAGCGGAGCAGGAGTCCTCGGCCTTTGGGCATTCTAGTTGAACGACTGTCGC |
| Cy5-c12 | Cy5-AGAGAACCTGGG |
| FAM-c12 | FAM-AGAGAACCTGGG |
| 5HS-27A-c12 | HS-AAAAAAAAAAAAAAAAAAAAAAAAAAAAGAGAACCTGGG |
| 17-3-6T12 | TCAACTGCCTGGTGATAAAACGACACTACGTGGGAATCTACTATGGCGGCTCTTCTTTTTTCCCAGGTTCTCT |
| 17-1 | ATCACCCAAACCCTCAATCTTTTACATTCCTAAGTCTGAAACATTACAGCTTGCTACACGAGAAGAGCCGCCATAGTA |
| 17-2 | ATCACCCAAACCCTCAATCTTTTTATCACCAGGCAGTTGACAGTGTAGCAAGCTGTAATAGATGCGAGGGTCCAATAC |
| 17-3 | ATCACCCAAACCCTCAATCTTTTTCAACTGCCTGGTGATAAAACGACACTACGTGGGAATCTACTATGGCGGCTCTTC |
| 17-4 | ATCACCCAAACCCTCAATCTTTTTTCAGACTTAGGAATGTGCTTCCCACGTAGTGTCGTTTGTATTGGACCCTCGCAT |
| H1-CHA | AGATTGAGGGTTTGGGTGATTTTTTTCAACATCAGTCTGATAAGCTACCATGTGTAGATAGCTTATCAGACT |
| H2-CHA | AGATTGAGGGTTTGGGTGATTTTTTTAAGCTATCTACACATGGTAGCTTATCAGACTCCATGTGTAGA |
| H1-CHA-FAM | AGATTGAGGGTTTGGGTGATTTTTTTCAACATCAGT(**FAM**)CTGATAAGCTACCATGTGTAGATAGCTTATCAGACT |
| H2-CHA-TAMRA | AGATTGAGGGTTTGGGTGATTTTTTTAAGCTATCTACACATGGTAGCTTATCAGACT(**TAMRA**)CCATGTGTAGA |
| H1-CHA-FAM-BHQ1 | AGATTGAGGGTTTGGGTGATTTTTTTCAACATCAGT(**FAM**)CTGATAAGCTACCATGTGTAGATAGCTTATCAGACT(**BHQ1**) |
| miR-21 | UAGCUUAUCAGACUGAUGUUGA |
| DNA-21 | TAGCTTATCAGACTGATGTTGA |
| miR-141 | UAACACUGUCUGGUAAAGAUGG |
| miR-155 | UUAAUGCUAAUCGUGAUAGGGGU |
| miR-205 | UCCUUCAUUCCACCGGAGUCU |
| miR-122b | UGGAGUGUGACAAUGGUGUUUGA |

FAM: 6-Carboxyfluorescein; TR: Texas Red; TAMRA: Carboxytetramethylrhodamine; BHQ1: Black Hole Quencher 1.

# Supplementary Data


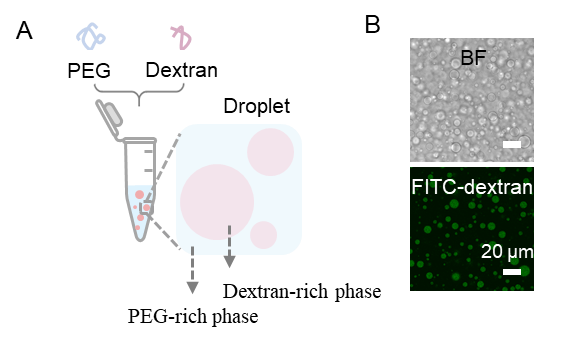


**Figure S1.** The droplet formation in the PEG/dextran system. A) Scheme showing the global PEG-rich phase and local dextran-rich phase. B) Confocal fluorescence images of the microdroplets prepared by mixing 1% dextran 70k and 10% PEG 20k dopped with 0.01% FITC-dextran 70k. Scale bar: 20 µm. The green fluorescence from the droplets indicated that the droplet phase was rich in dextran.


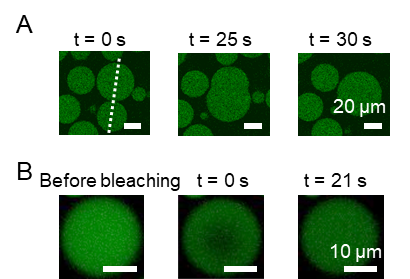


**Figure S2.** The liquid-like properties of ATPS droplets. A) Fusion events showing the droplets grew in size via collision with one other. B) Fluorescence recovery after photobleaching (FRAP) experiments showing immediate recovery of the fluorescence intensity. These demonstrated that the ATPS droplets have liquid-like properties.


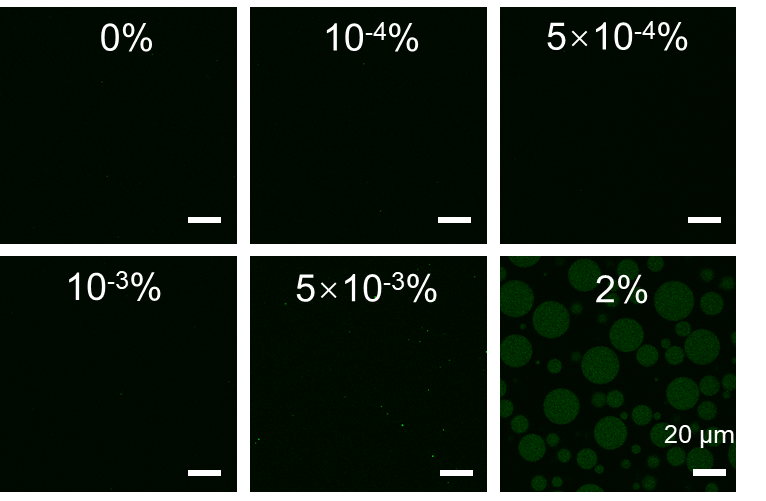


**Figure S3.** CLSM images of ATPS with 10% PEG 20k as a function of dextran 70k concentration from 10^-4^% to 5%. FITC-labeled dextran (70k, final 43 nM) was introduced into the system to characterize the droplets. When the dextran concentration was 5 × 10^-3^%, green fluorescent dots appeared, indicating the production of droplets. As the dextran concentration increased, the droplets became larger and more numerous. Scale bars are 20 µm.


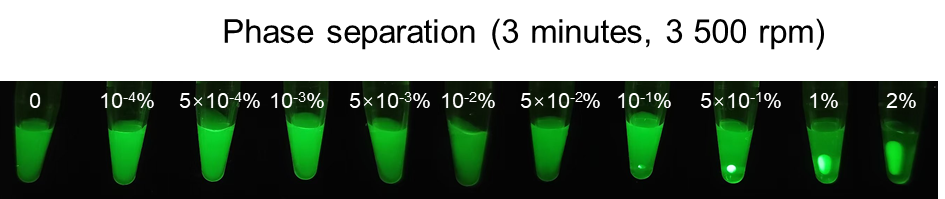


**Figure S4.** Images of ATPS with 10% PEG 20k as a function of dextran 70k concentration from 10^-4^% to 2% after centrifugation (3 min, 3 500 rpm). FITC-labeled dextran (70k, 0.01%) was introduced into the system. Bright spots were formed when the dextran concentration was 0.1%, which indicated macroscopic phase separation. However, no phase separation happened when the dextran concentration was below 0.1%. The droplet size in the PEG/dextran system depends on dextran concentration.


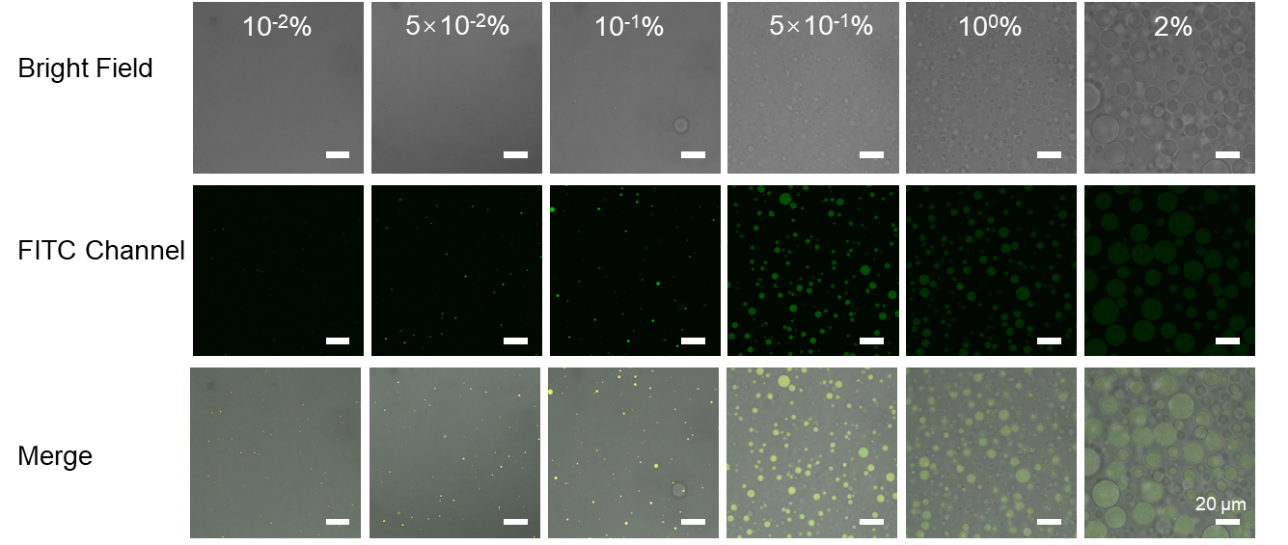


**Figure S5.** CLSM images of droplets corresponding to the bottom row in Figure 1B. TDN was 50 nM. FITC-dextran was employed as a fluorescent tracer to visualize green fluorescence localized within droplets under the FITC channel. The merge channel showed the overlapping dextran-rich droplets (green fluorescence) and the Cy5-labeled DNA (red fluorescence). The concentration of PEG 20k was 10%. Scale bars are 20 µm.


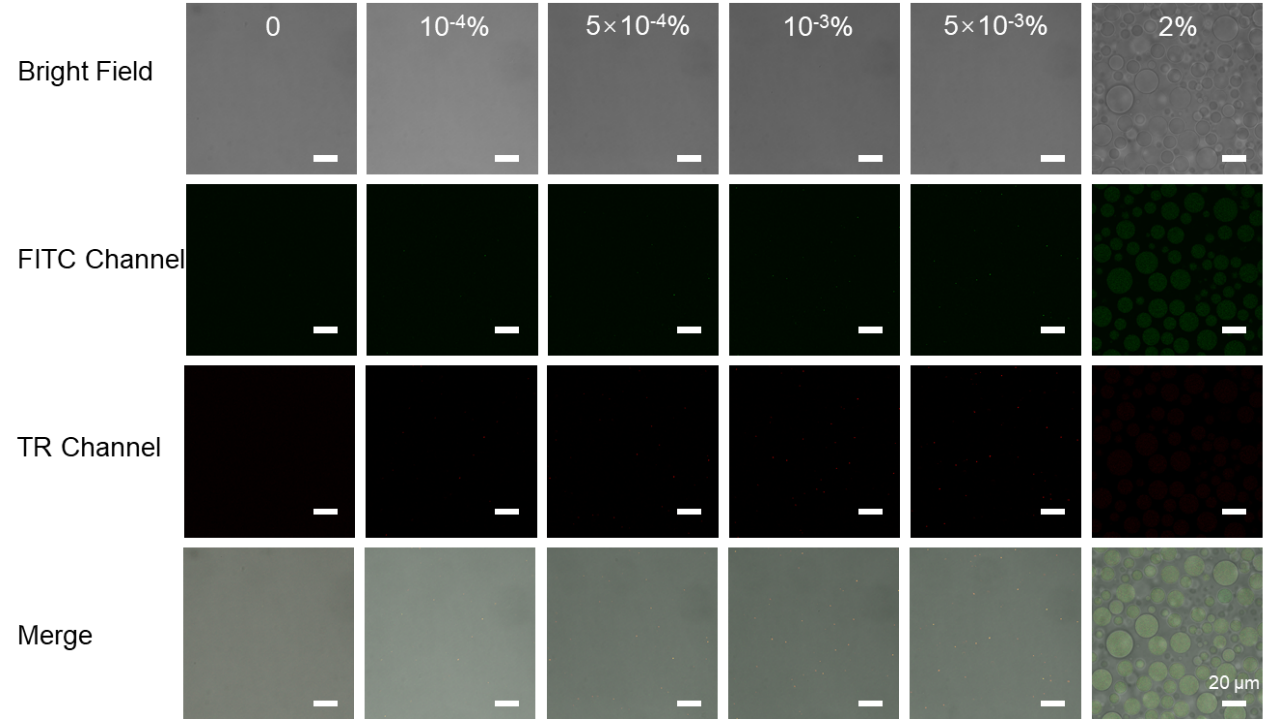


**Figure S6.** CLSM images showing Texas red-labeled TDN enrichment into dextran-rich droplets under varied dextran 70k concentrations mixed with 10% PEG 20k in buffer A. TDN was 50 nM. Scale bars are 20 µm.


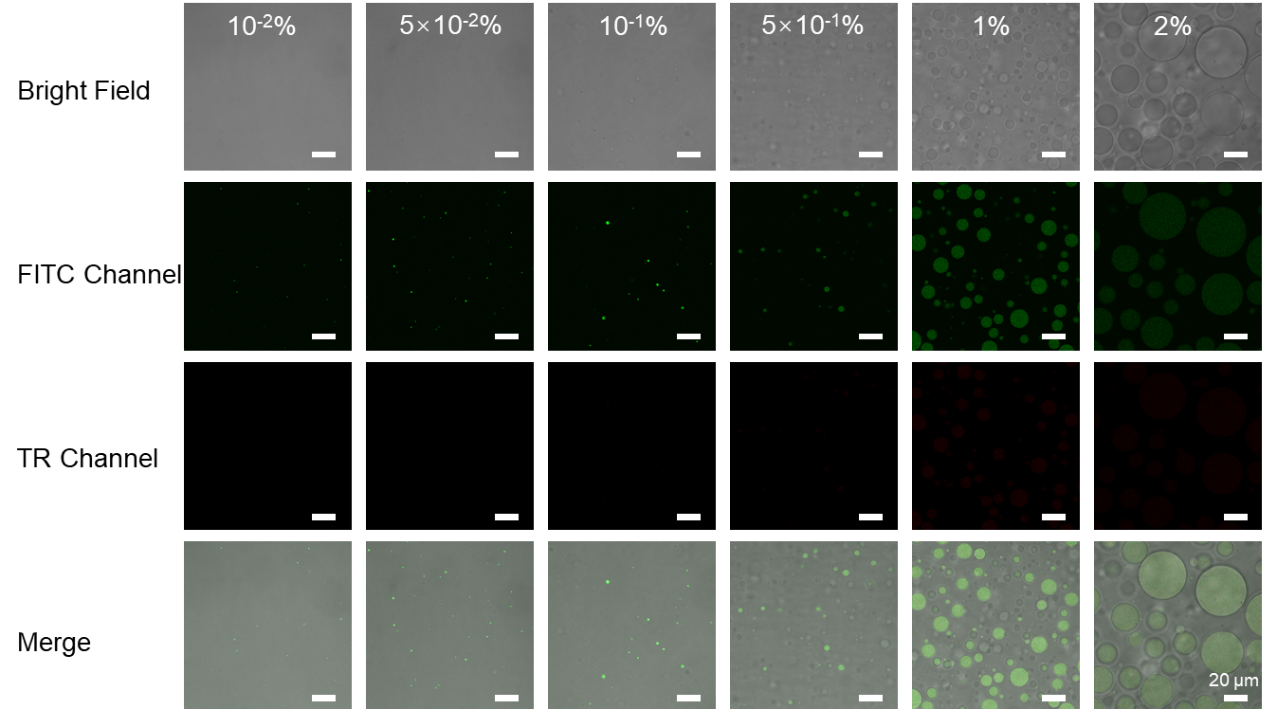


**Figure S7.** CLSM images showing Texas red-labeled ssDNA enrichment. The concentration of ssDNA was 50 nM. Weak fluorescence intensity was observed, which meant that the ssDNA had a weak partition into the dextran-rich droplets.


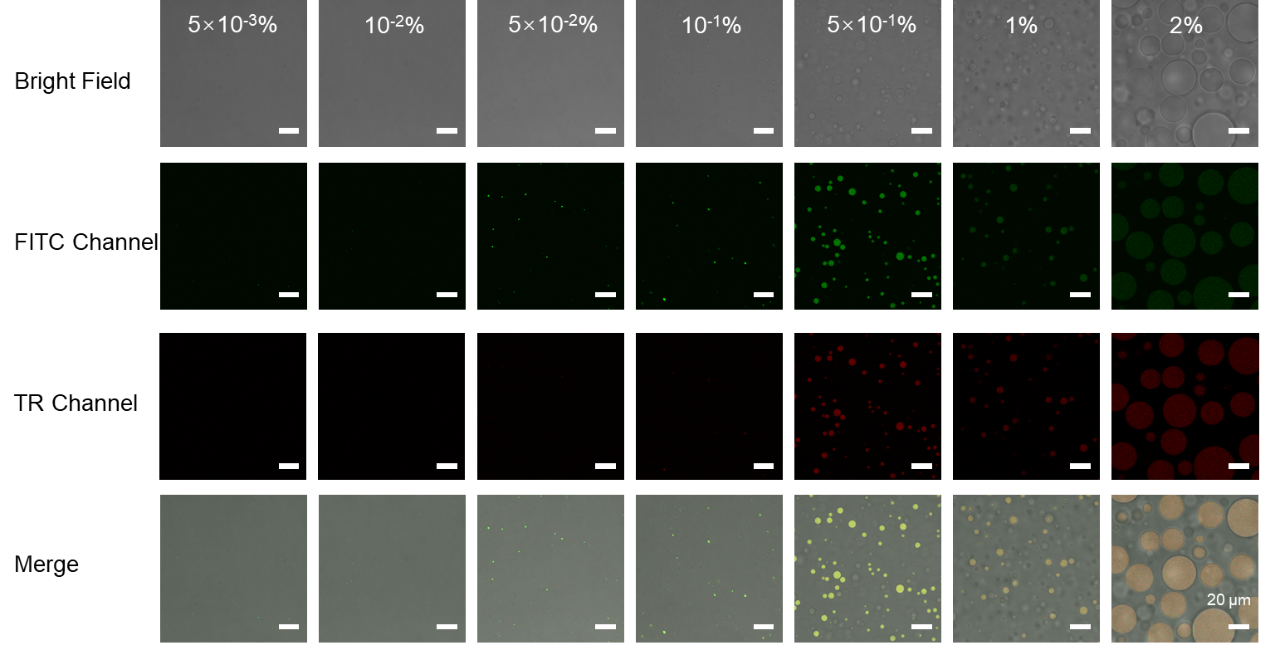


**Figure S8.** Confocal fluorescence images of ssDNA partition in dextran-rich droplets under different concentrations of dextran 70k mixed with 10% PEG 20k in buffer A. ssDNA was labeled with Cy5 and the concentration was 200 nM. Increasing ssDNA concentration led to greater DNA accumulation in larger droplets. Scale bars are 20 µm.


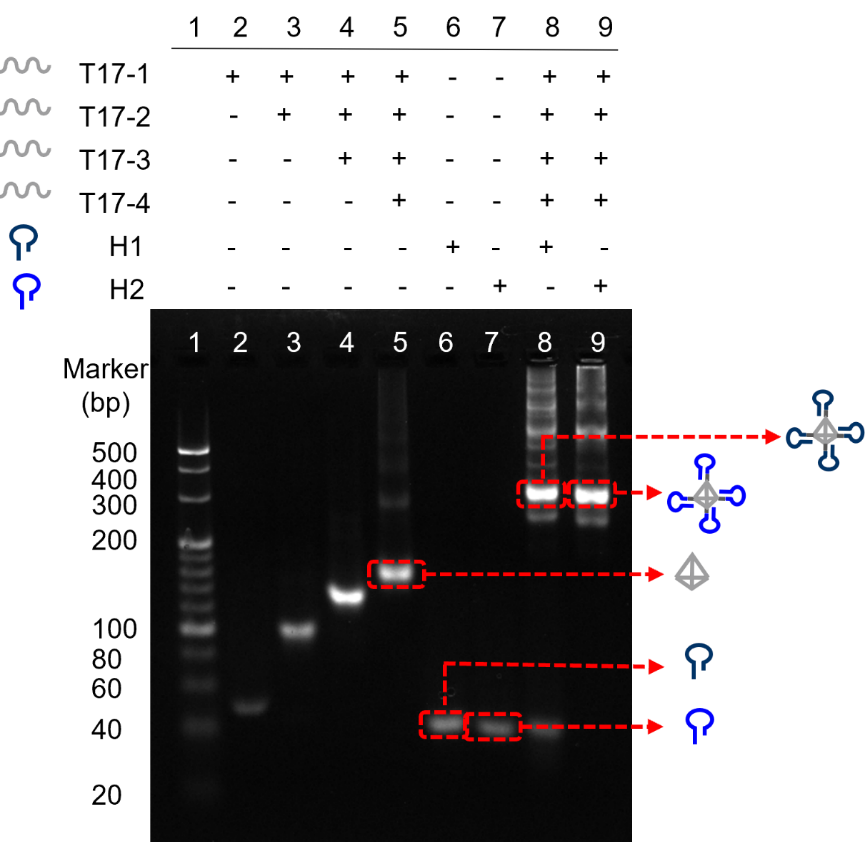


**Figure S9.** Agarose gel electrophoresis (3%) was conducted at a constant voltage of 120 V for 45 min to analyze the TDN. Lanes 1-3 display bands corresponding to TDN building blocks with one, two, and three strands, respectively. Lane 5 shows the formation of TDNs with four single-stranded DNAs. After introducing the tethered hairpins, lanes 8 and 9 exhibit similar bands, indicating the successful preparation of hairpin-modified DNA TDNs.


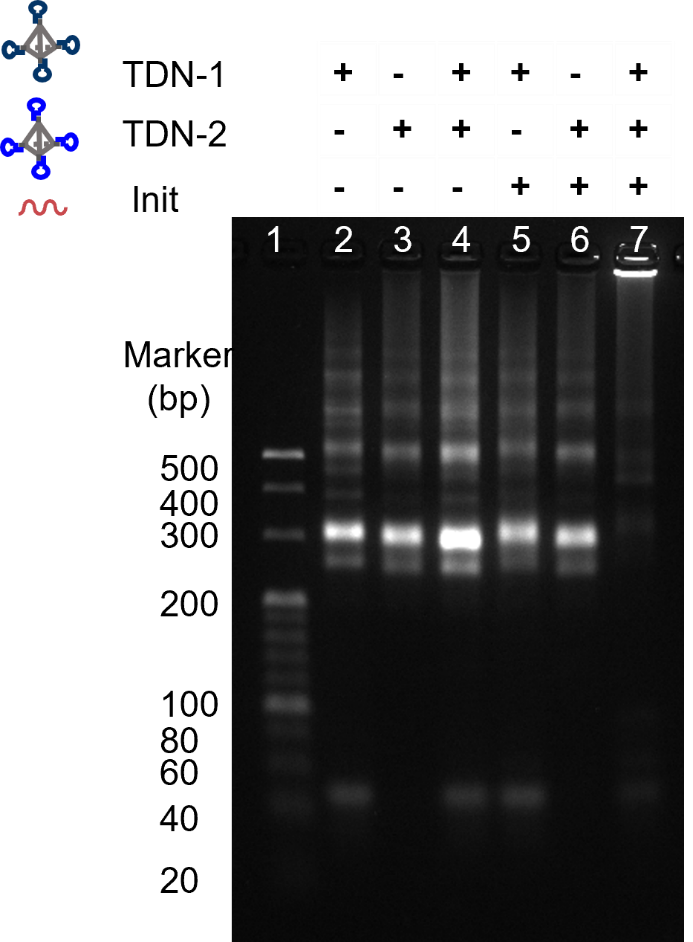


**Figure S10.** Agarose gel electrophoresis (3%) analysis of the TDN superstructures at a constant voltage of 120 V for 45 min. Lane 1 is the 20 bp marker. Lane 2 and 3 are TDN-1 and TDN-2. No TDN aggregates were observed when only one type of TDN was mixed with the initiator (Lines 4 and 5). However, the accumulation of TDN products in the well occurred in the presence of all three DNAs (Line 6), indicating the formation of TDN superstructures.


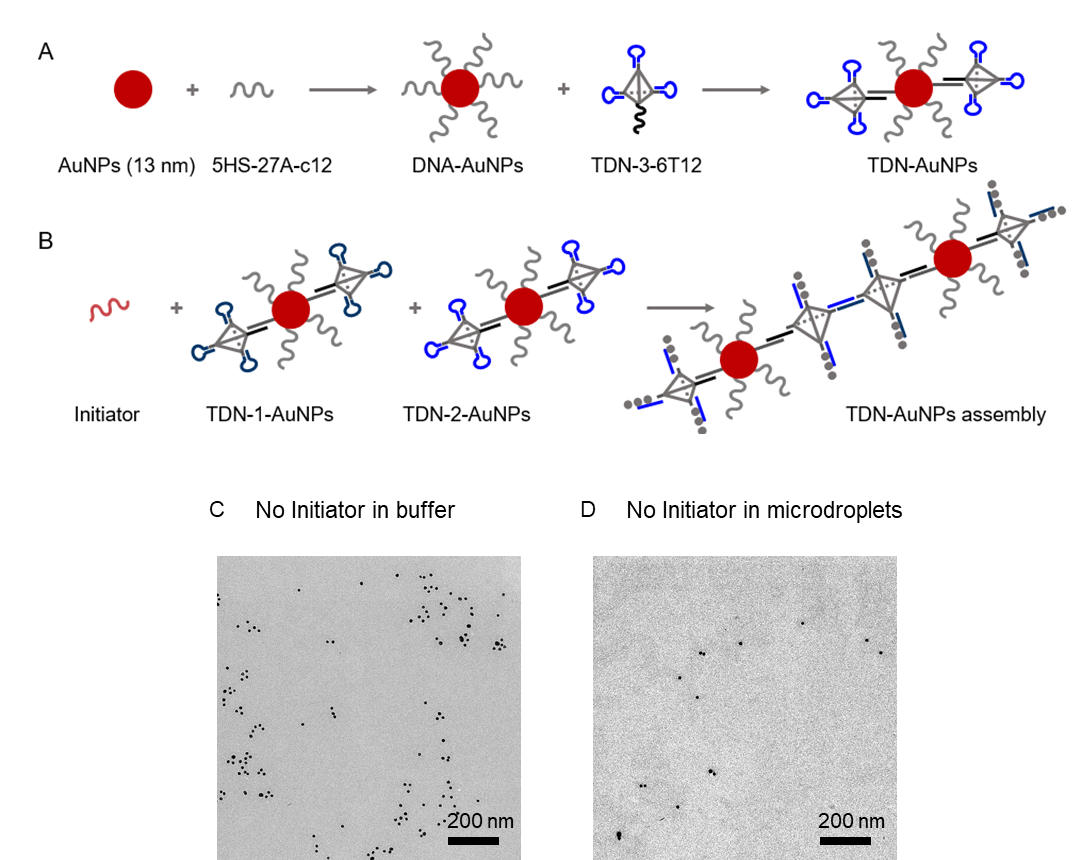


**Figure S11.** Cartoon of A) Conjugation process of AuNPs and TDN and B) TDN-AuNPs assembly with the Initiator. TEM images of TDN-AuNPs before adding Initiator in C) Buffer A and D) microdroplets (10% PEG 20k and 1% dextran 70k). No TDN-AuNP assembly was observed, which indicated that the aqueous two-phase system (ATPS) environment exhibited no discernible influence on the colloidal stability of TDN-AuNPs. The concentration of TDN-AuNPs utilized was 1 nM. The Initiator was 20 nM.


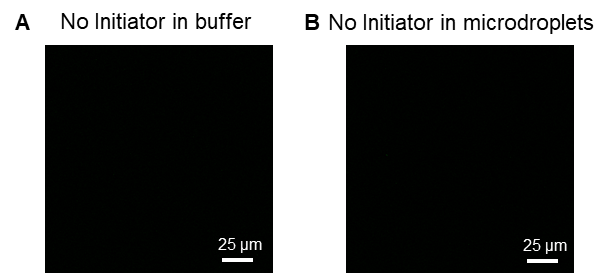


**Figure S12.** The confocal laser scanning fluorescence microscopy (CLSM) images show TDN in A) buffer and B) PEG/dextran. No fluorescence was observed in either image, indicating that the TDNs were too small to be visualized in the absence of initiators. The concentration of TDNs used was 100 nM.


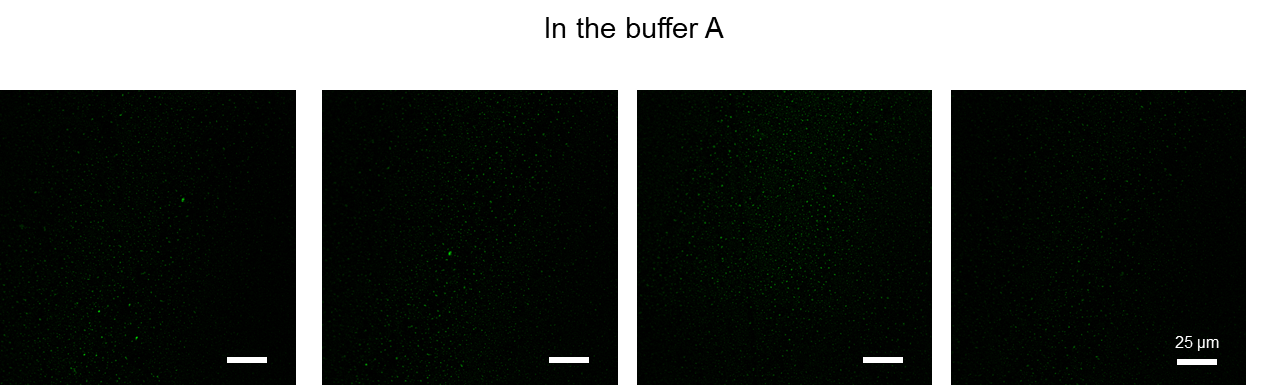


**Figure S13.** Typical CLSM images of TDN assemblies in buffer A for analyzing size distribution by Image J. DNA TDNs and initiator are 100 nM. The statistical results are shown in Figures 2E,F. DNA was stained by SYBR Green I. Only small dots were observed, which demonstrated limited TDN assembly.


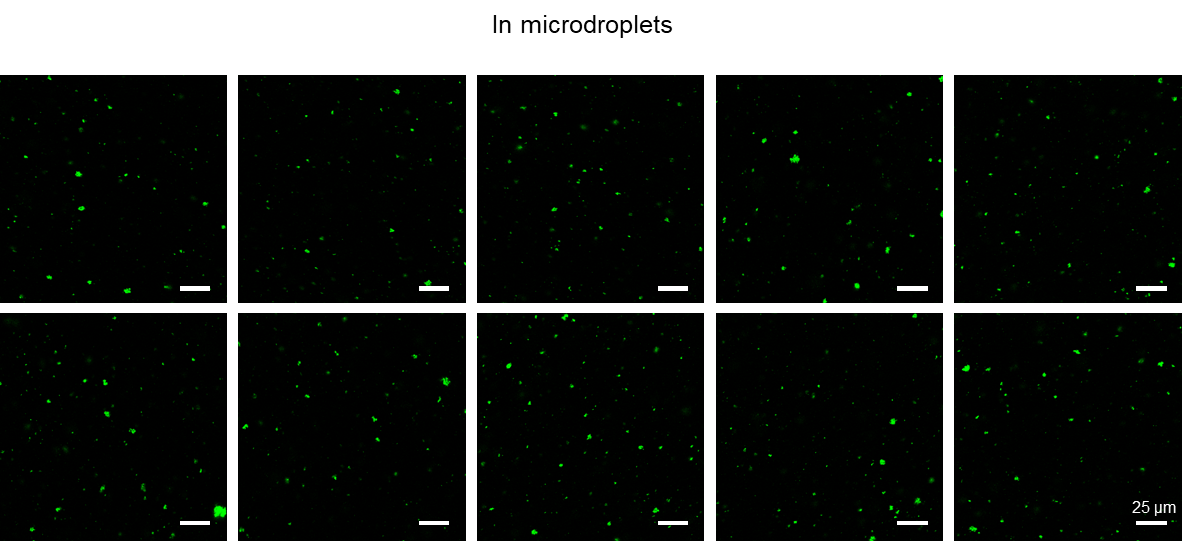


**Figure S14.** Typical CLSM images of TDN assemblies in microdroplets for statistic analysis (Figures 2E,F). PEG 20k was 10%, and dextran 70k was 1%. DNA TDNs and initiator were 100 nM. The assemblies were stained by SYBR Green I.


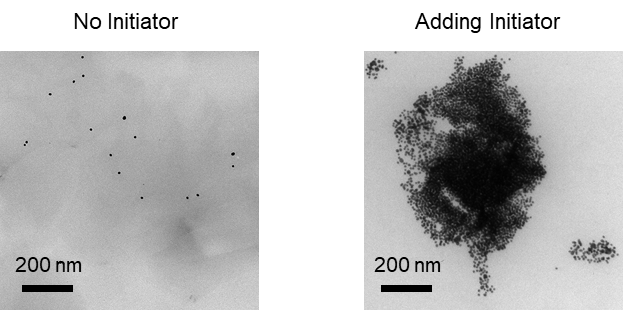


**Figure S15.** TEM images of TDN assemblies in 0.05% sub-micron droplets correlate with Figure 3A. DNA TDNs and Initiator were 5 nM. The assembled architectures of TDN-AuNPs exhibited increased dimensional scales and enhanced structural compactness.


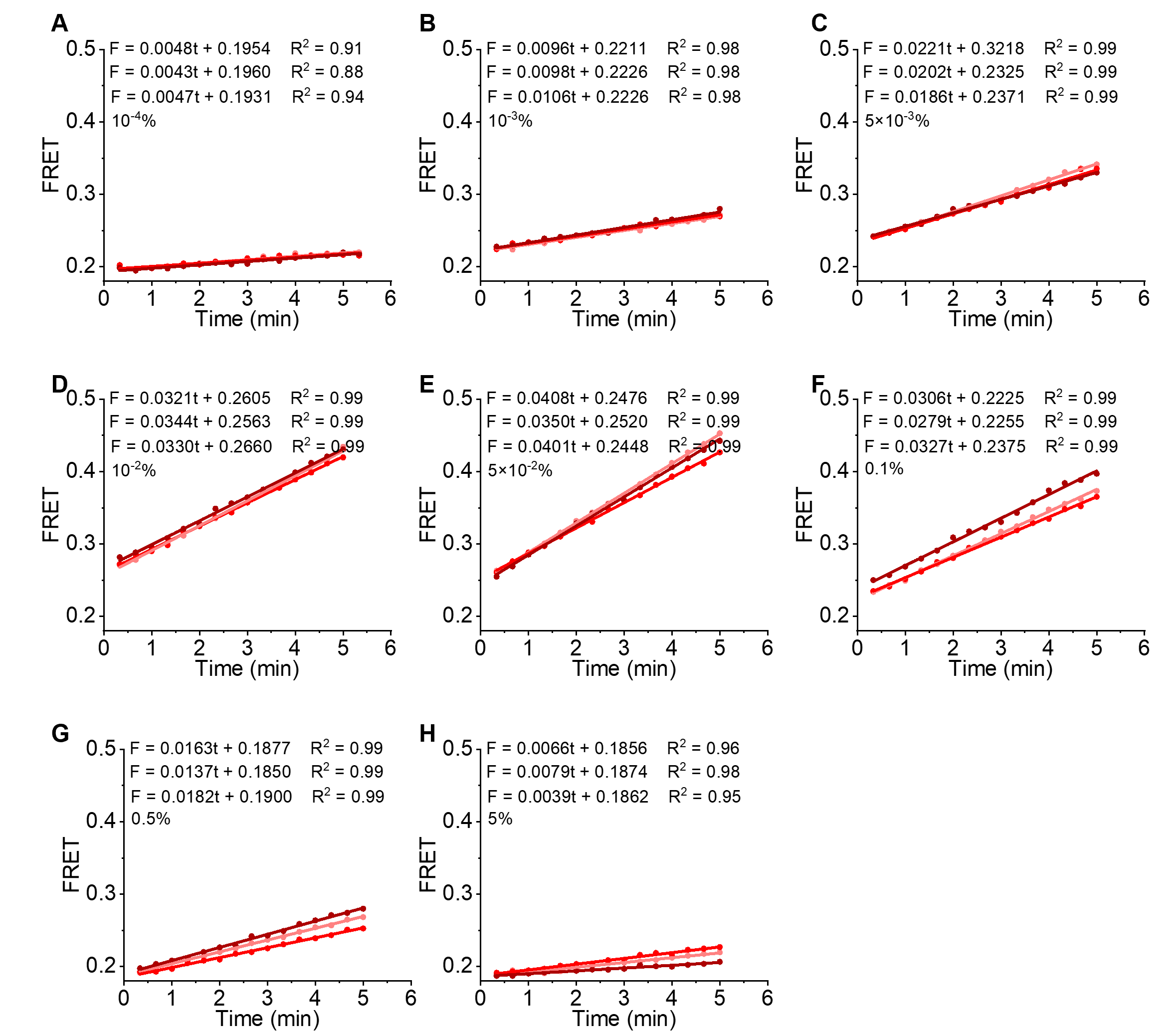


**Figure S16.** The initial rates of TDN-17 CHA reactions at different dextran 70k concentrations with a constant 10% PEG 20k. The concentrations of dextran 70k were: A) 10^-4^%, B) 10^-3^%, C) 5 × 10^-3^%, D) 10^-2^%, E) 5 × 10^-2^%, F) 0.1%, G) 0.5% and H) 5%. Each reaction was repeated 3 times. Initiator (5 nM) was introduced a mixture of 5 nM TDN-1-FAM and TDN-2-TAMRA in Buffer A. Firstly, equilibrate the mixture for three minutes, then the initiator was added. The reaction rate first increased even with only 0.01% dextran 70k D). However, the rate decreased with increasing dextran concentrations.


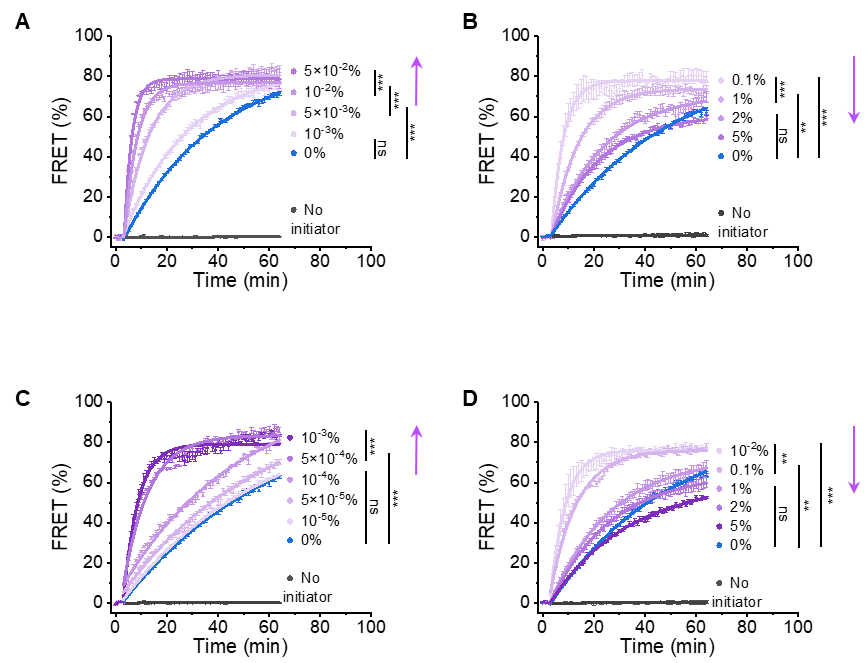


**Figure S17.** Kinetics of DNA assembly by FRET as a function of dextran 40k concentrations from A) 10^-3^% to 10^-2^% and B) 0.1% to 5% in 10% PEG. And the reaction kinetics of dextran 150k from C) 10^-5^% to 10^-2^% and D) 0.1% to 5% in 10% PEG. With increasing dextran, the reaction rate first increases to a maximum and then decreases close to that in a pure PEG environment. The critical concentrations were 0.05% for 40k and 10^-3^% for 150k. Data are shown as the mean ± SD (*n* = 3). Statistical signiﬁcance was calculated via one-way ANOVA with Tukey’s test: ***p* < 0.01, and ****p* < 0.001; “ns” indicated not signiﬁcant.


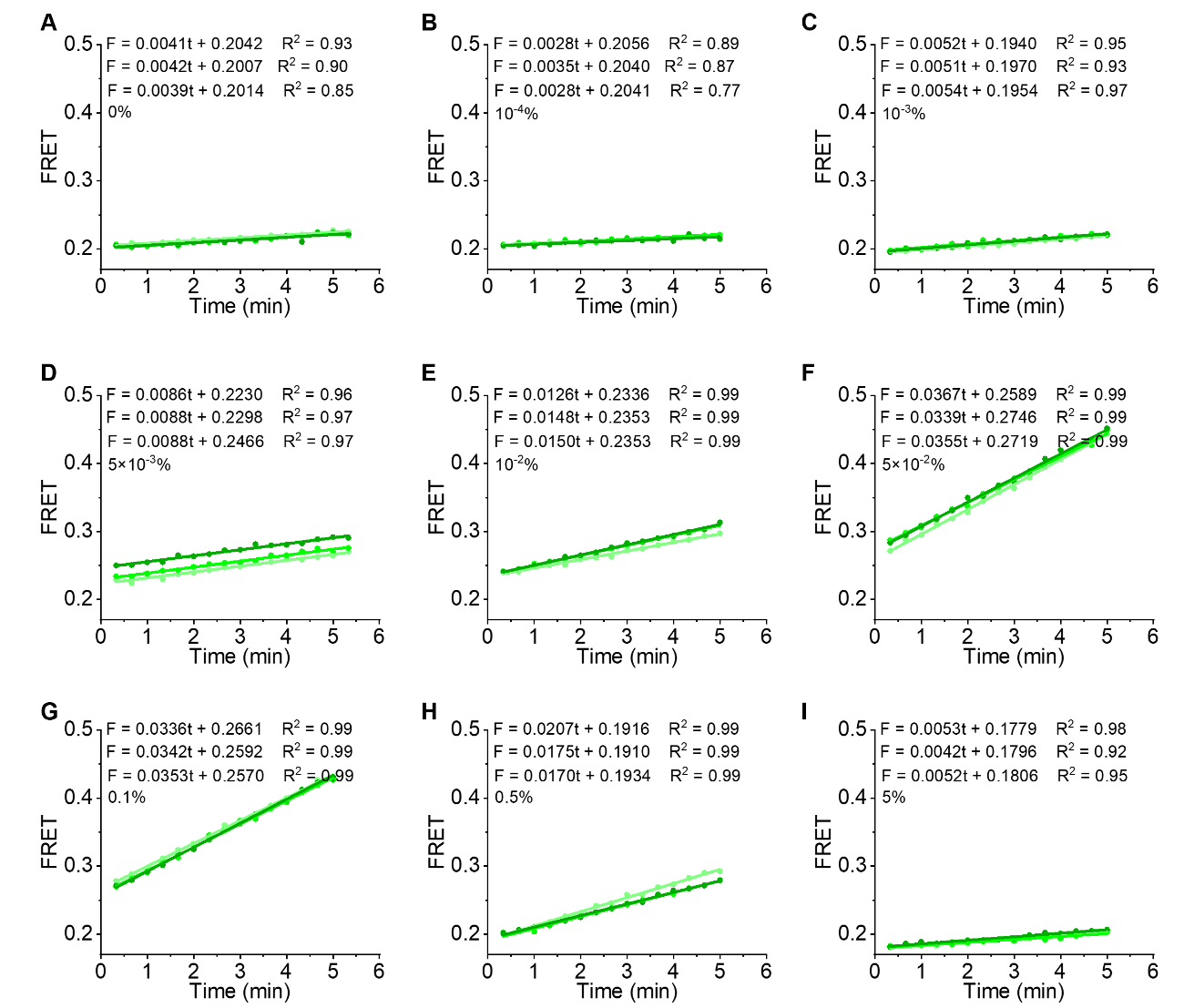


**Figure S18.** First five minutes reaction kinetics curves of TDN-17 at different dextran 40k concentrations with a constant 10% PEG 20k. Dextran 40k concentrations were A) 0%, B) 10^-4^%, C) 10^-3^%, D) 5 × 10^-3^%, E) 10^-2^%, F) 5 × 10^-2^%, G) 0.1%, H) 0.5% and I) 5%. Initiator (5 nM) was introduced into a mixture of 5 nM TDN-1 and TDN-2 in Buffer A. Dextran 40k has a similar trend with 0.05% critical concentrations. Data are shown as the mean ± SD (*n* = 3).


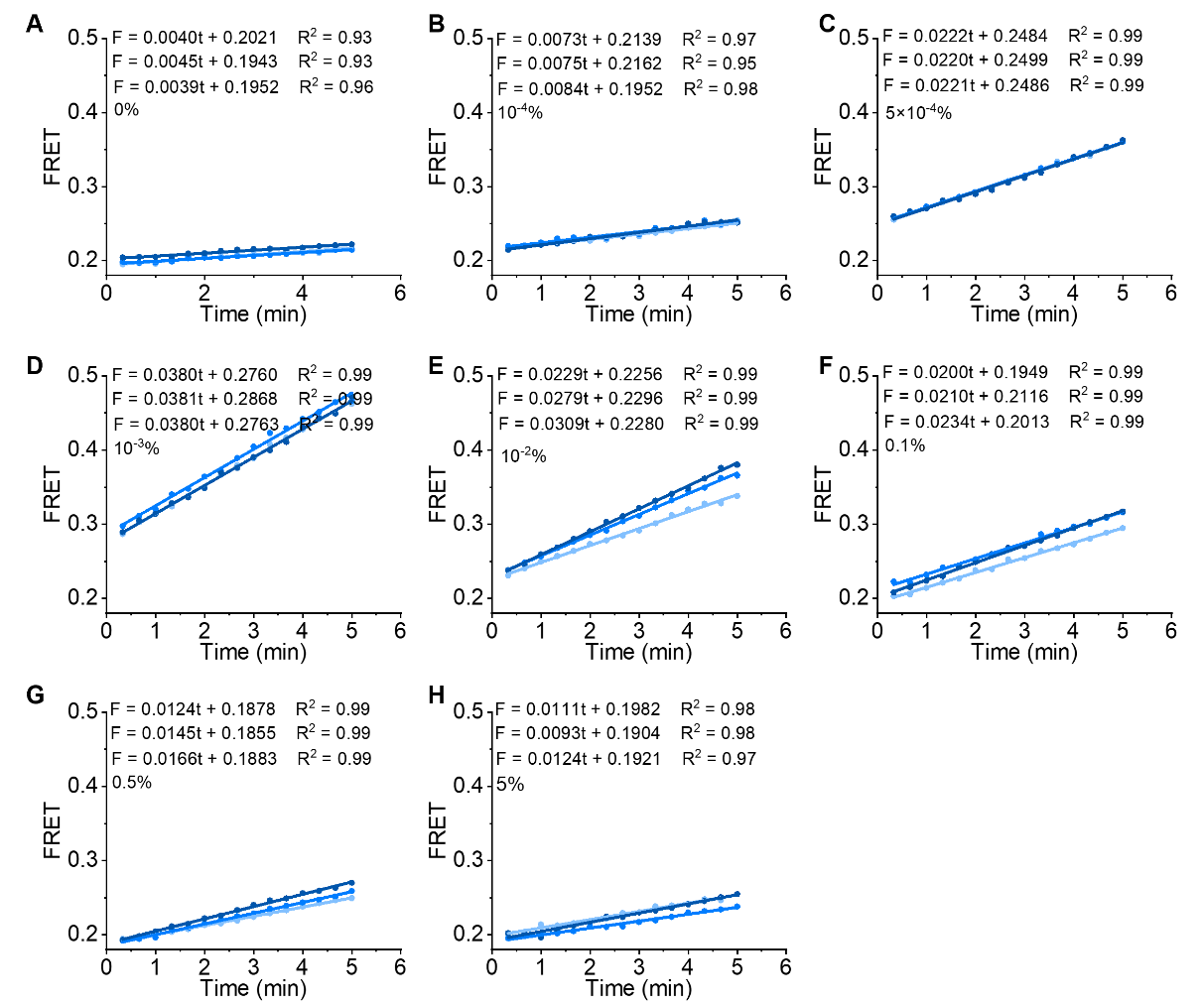


**Figure S19.** The FRET curves of TDN-17 CHA reactions at different dextran 150 concentrations with a constant 10% PEG 20k for the first five minutes. The concentrations of dextran 150k from A-H) were 0%, 10^-4^%, 5 × 10^-4^%, 10^-3^%, 10^-2^%, 0.1%, 0.5%, and 5%. An equal matrix of Initiator, TDN-1, and TDN-2 was reacted (5 nM). The trend was similar to dextran 40k and 70k. The critical concentration was 10^-3^%, which was lower due to stronger dextran-DNA interaction. Data are expressed as the mean ± SD (*n* = 3).


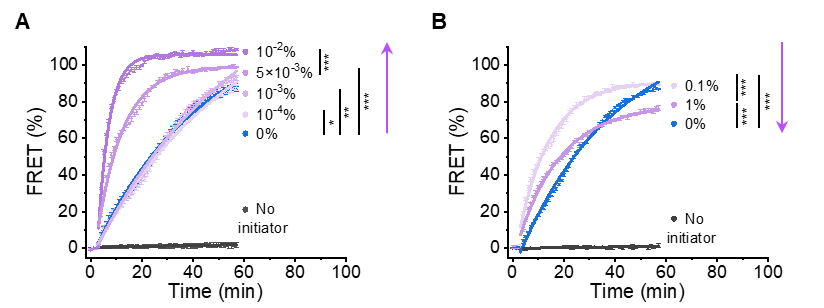


**Figure S20.** Promotional effect of increasing TDN concentration. Dextran 70k concentrations were from A) 10^-4^% to 10^-2^% and B) 0.1% to 1%. The concentration of TDN and initiator was adjusted to 20 nM, but the optimal dextran did not change. This phenomenon suggests that this critical concentration is an intrinsic property of the mixed polymer system. Data are shown as the mean ± SD (*n* = 3). Statistical signiﬁcance was calculated via one-way ANOVA with Tukey’s test: ***p* < 0.01, and ****p* < 0.001; “ns” indicated not signiﬁcant.


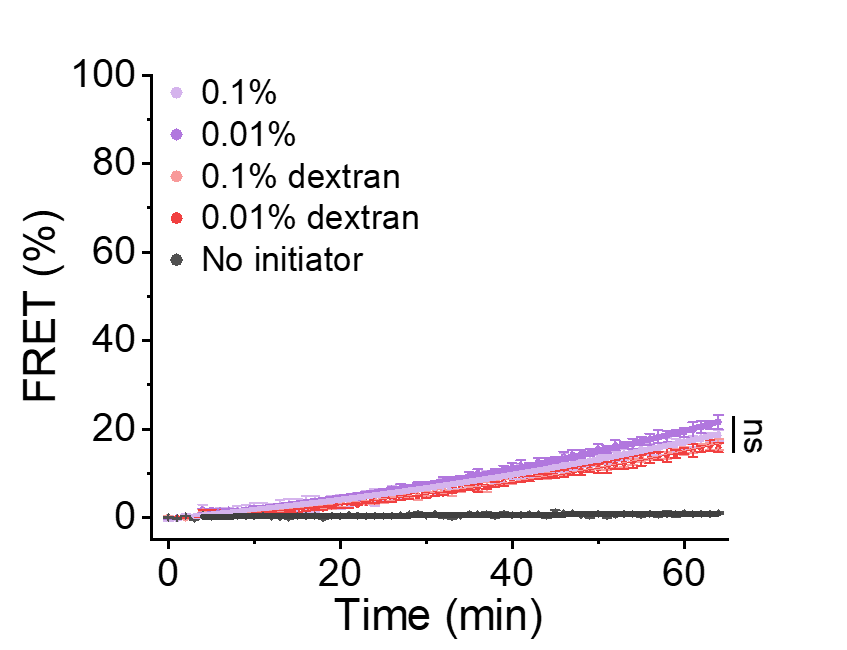


**Figure S21.** The effect of PEG in the dextran-rich phase. We collected the bottom dextran-rich phase after the centrifugation of the PEG and dextran mixtures. The reaction kinetics were similar to that in the pure dextran solution. This indicated that the locally crowded dextran environment may not be the direct reason for the accelerated reactions. Data are shown as the mean ± SD (*n* = 3). Statistical signiﬁcance was calculated via one-way ANOVA with Tukey’s test: “ns” indicated not signiﬁcant.

**
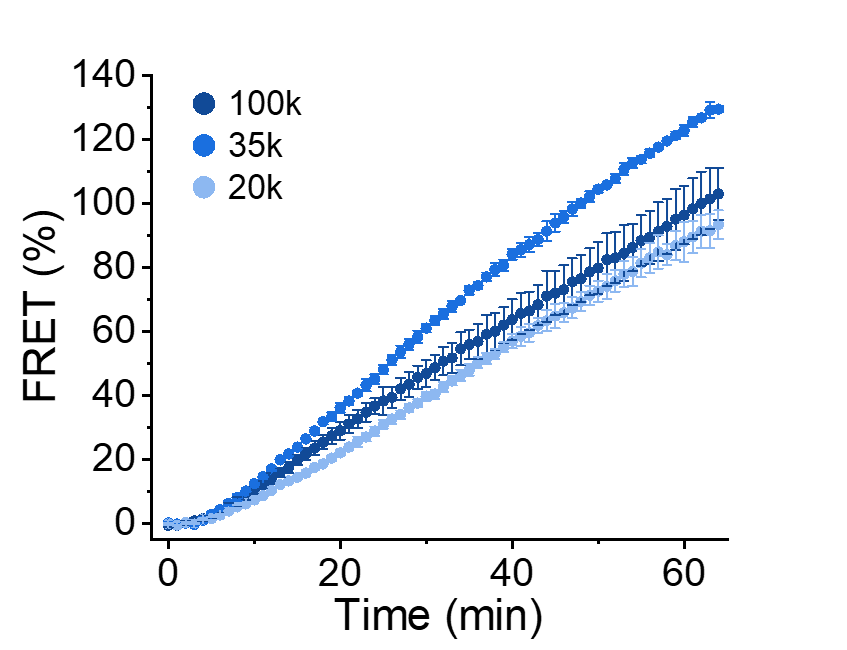
**

**Figure S22.** Kinetics of DNA assembly as a function of PEG molecular weights (10%, 35 and 100k). The reaction kinetics were faster than PEG 20k. However, PEG 35k and 100k exhibit markedly elevated viscosity, which is not convenient for subsequent experiments. Data are expressed as the mean ± SD (*n* = 3).


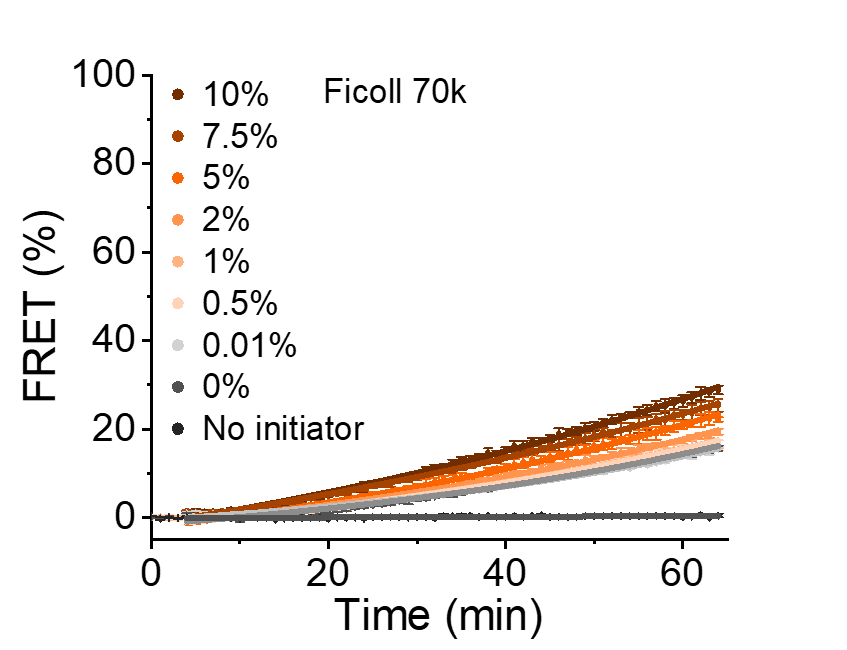


**Figure S23.** The effect of Ficoll 70k concentrations on TDN CHA reaction. Ficoll only slightly promoted the reaction, which was similar to dextran. Ficoll of different concentrations (0%, 0.01%, 0.5%, 1%, 2%, 5%, 7.5%, and 10%) was mixed with Buffer A. Data are shown as the mean ± SD (*n* = 3).


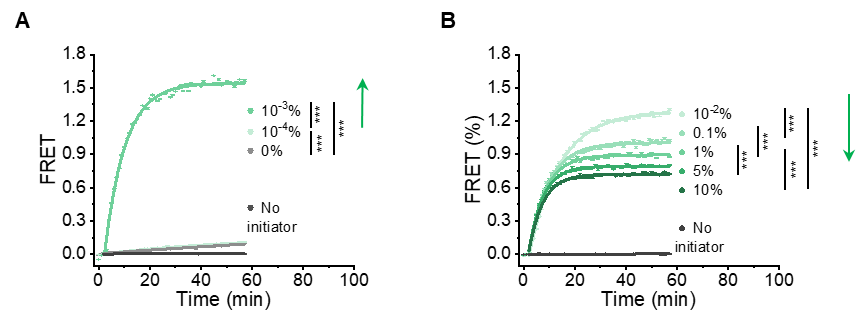


**Figure S24.** Effect of positively charged polymers on the reaction. Positively charged polymer (poly (diallyl dimethylammonium chloride), PDDA) has a stronger electrostatic interaction with nucleic acids. It accelerates the TDN assembly at a low concentration, which could screen the charge repulsion between negatively charged DNAs A). However， PDDA inhibits it at higher concentrations B). Excess PDDA introduces additional repulsion forces of similar charges. Data are shown as the mean ± SD (*n* = 3). Statistical signiﬁcance was calculated via one-way ANOVA with Tukey’s test: ****p* < 0.001.


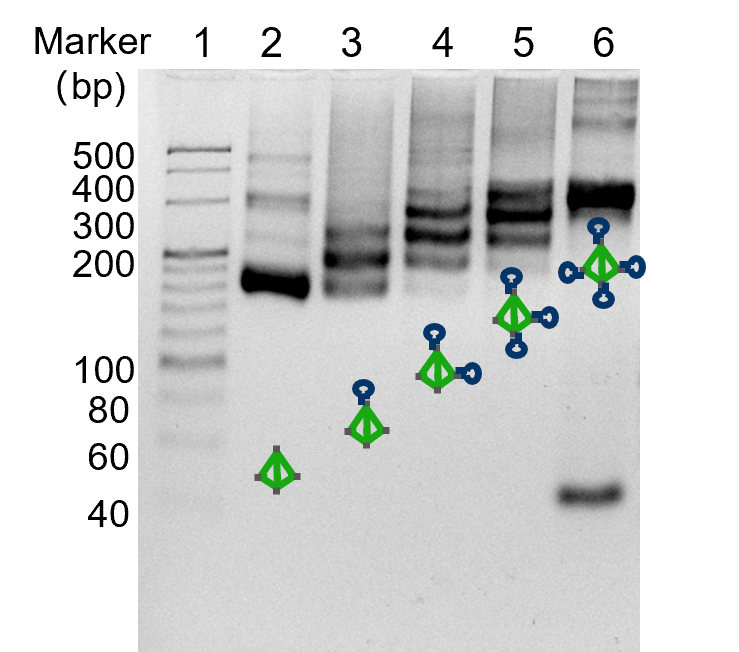


**Figure S25.** Agarose gel electrophoresis (3%) analysis of TDN-17 with different numbers of hairpins.


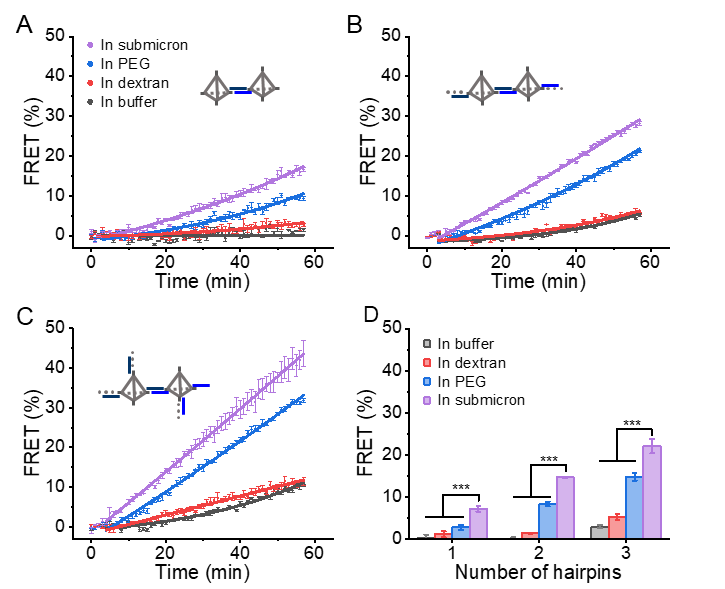


**Figure S26.** The effect of TDN-17 with different numbers of molecular hairpins. A) One hairpin, B) two hairpins, and C) three hairpins. D) FRET value at the 30th minute in four different environments (buffer, dextran, PEG, and PEG/dextran). The CHA reaction was more facilitated by the mixed crowding environment than the single polymer. Data are shown as the mean ± SD (*n* = 3). Statistical signiﬁcance was calculated via one-way ANOVA with Tukey’s test: ****p* < 0.001.


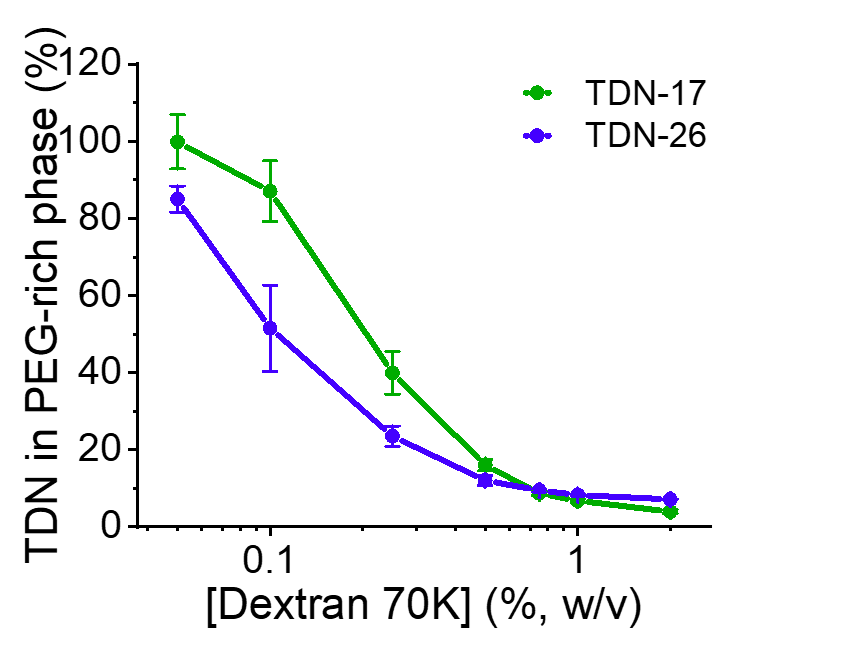


**Figure S27.** The partition of TDN-17 and TDN-26 in the PEG-rich phase as a function of a dextran concentration. TDN preferred to dextran-rich phase with increasing dextran concentration. The larger the TDN size, the more pronounced this trend becomes. Data are shown as the mean ± SD (*n* = 3).


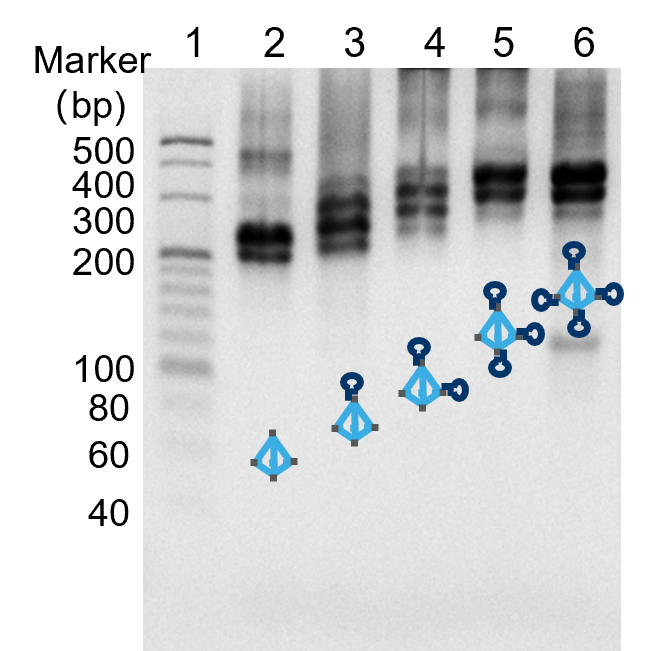


**Figure S28.** Agarose gel electrophoresis (3%) analysis of TDN-26 with different numbers of hairpins.


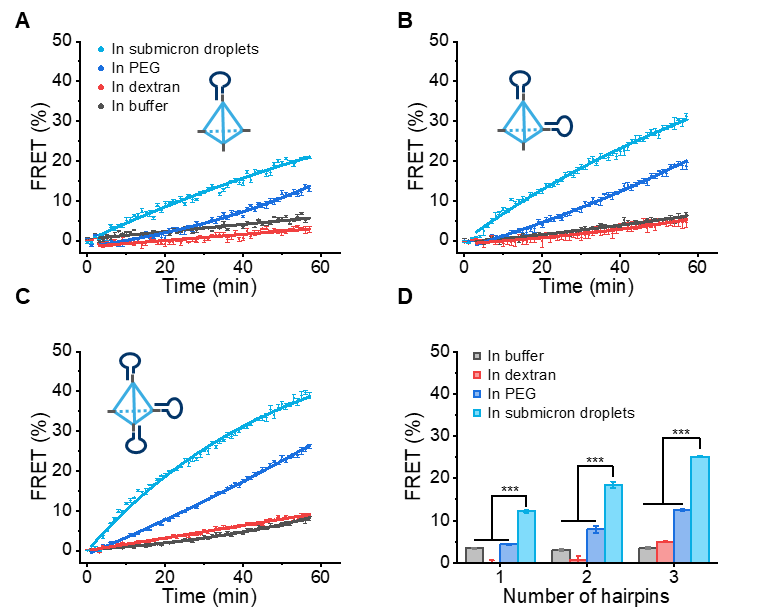


**Figure S29.** The effect of TDN-26 with different numbers of molecular hairpins. A) One hairpin, B) two hairpins, and C) three hairpins. D) FRET value at the 30th minute in four different environments (buffer, dextran, PEG, and PEG/dextran). The CHA reaction was more facilitated by the mixed crowding environment than the single polymer. Data are shown as the mean ± SD (*n* = 3). Statistical signiﬁcance was calculated via one-way ANOVA with Tukey’s test: ****p* < 0.001.

**
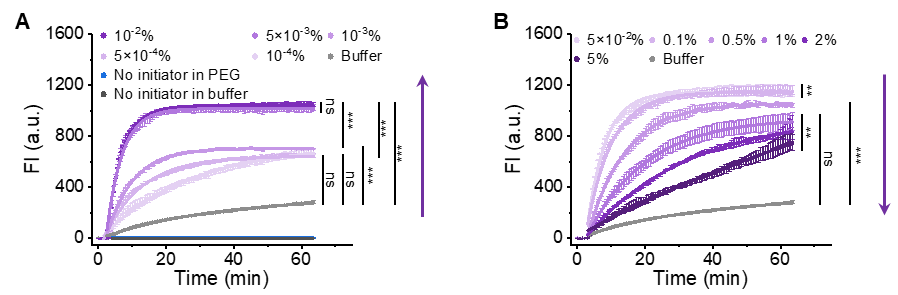
**

**Figure S30.** Kinetics of target detection by FRET (FAM and BHQ1 pair) as a function of dextran 70k concentrations from A) 5 × 10^-4^% to 10^-2^% and B) 5 × 10^2^% to 5%. The concentration of TDN was 5 nM and the target was 5 nM. With increasing dextran, the detection rate first increases to the maximum and then decreases. The critical concentration were 0.01%. Data are expressed as the mean ± SD (*n* = 3). Statistical signiﬁcance was calculated via one-way ANOVA with Tukey’s test: ***p* < 0.01, and ****p* < 0.001; “ns” indicated not signiﬁcant.


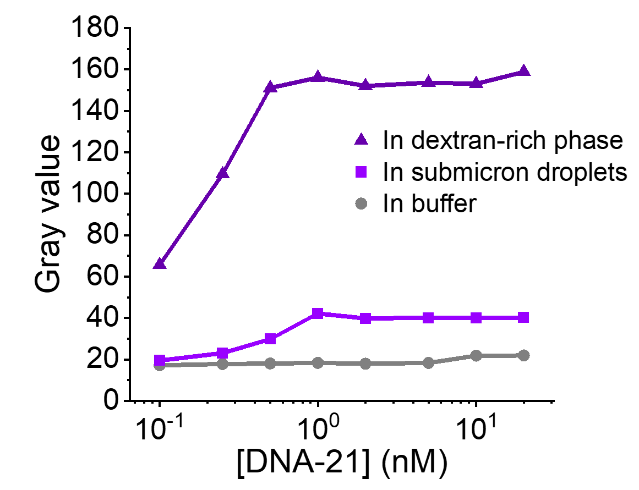


**Figure S31.** The gray value of images in Figure 6E. The fluorescence intensity was recorded with various concentrations of the target (0, 0.1, 0.25, 0.5, and 1 nM) in buffer A, 0.05% nanodroplets, and the dextran-rich phase. The reaction was accelerated by 0.05% nanodroplets and the fluorescence signal was amplified in the dextran-rich phase, which could not be observed in the buffer.
